# CD133^+^ endothelial-like stem cells restore neovascularization and promote longevity in progeroid and naturally aged mice

**DOI:** 10.1038/s43587-023-00512-z

**Published:** 2023-11-09

**Authors:** Shimin Sun, Yuan Meng, Mingying Li, Xiaolong Tang, Wenjing Hu, Weiwei Wu, Guo Li, Qiuxiang Pang, Wengong Wang, Baohua Liu

**Affiliations:** 1 Shenzhen Key Laboratory for Systemic Aging and Intervention (SKL-SAI), Guangdong Key Laboratory of Genome Stability and Human Disease Prevention; International Cancer Center, School of Basic Medical Sciences, Shenzhen University, Shenzhen, China; 2 Friedrich Schiller University, Jena, Germany; 3 Wannan Medical College, Wuhu, China; 4 School of Biomedical Sciences, Hunan University, Changsha, China; 5 Anti-aging & Regenerative Medicine Research Institution, School of Life Sciences, Shandong University of Technology, Zibo, China; 6 Department of Dermatology, Xiangya Hospital, Central South University, Changsha, China; 7 Department of Biochemistry and Molecular Biology, School of Basic Medical Sciences, Peking University Health Science Center, Beijing, China

**Keywords:** Ageing, Senescence, Ageing

## Abstract

The stem cell theory of aging dictates that a decline in the number and/or function of stem cells causes tissue degeneration and aging; however, it still lacks unequivocal experimental support. Here, using lineage tracing and single-cell transcriptomics, we identify a population of CD133^+^ bone marrow-derived endothelial-like cells (ELCs) as potential endothelial progenitor cells, which contribute to tubular structures in vitro and neovascularization in vivo. We demonstrate that supplementation with wild-type and young ELCs respectively restores neovascularization and extends lifespan in progeric and naturally aged mice. Mechanistically, we identify an upregulation of farnesyl diphosphate synthase (FDPS) in aged CD133^+^ ELCs—a key enzyme in isoprenoid biosynthesis. Overexpression of FDPS compromises the neovascularization capacity of CD133^+^ ELCs, whereas FDPS inhibition by pamidronate enhances neovascularization, improves health measures and extends lifespan in aged mice. These findings highlight stem cell-based strategies for the treatment of progeria and age-related pathologies.

## Abstract (web summary)

Sun et al. identify a stem cell population of CD133^+^ endothelial-like cells (ELCs) that contribute to neovascularization. ELCs become dysfunctional with age, but ELC supplementation or pamidronate treatment to counter ELC aging promotes longevity.

## Main

Tissue degeneration is a typical characteristic of aging^1^. A small population of stem cells or progenitors that reside in specific tissues, called tissue stem cells (TSCs), are capable of self-renewal and differentiation into tissue-specific cells, thus maintaining tissue homeostasis^2^. The hematopoietic system is one representative of high-turnover tissues with a high demand on TSCs, that is, hematopoietic stem cells (HSCs); a single HSC can repopulate the whole blood system^3,4^. Mesenchymal stem cells (MSCs) and endothelial progenitor cells (EPCs) are two other prominent types of TSC in adult bone marrow (BM)^5^. While MSCs can be easily expanded in vitro and show great clinical application potential^6,7^, the nomenclature and origination of EPCs are still hotly debated^8–10^. Asahara et al. first determined the existence of human peripheral blood endothelial progenitor cells^11^, which promote vascular regeneration^12^. Upon endothelium injury, cytokines and growth factors, such as VEGF, SDF-1, G-CSF and estrogen, mobilize EPCs from the BM to the peripheral circulation, which then seed at the injury sites and mediate neovascularization^8,13^. Typical EPC enrichment usually requires 1–4 weeks of in vitro culture. According to the culture time, two main types of EPC can be obtained: myeloid angiogenic cells (MACs) require a 4–7-day culture, while endothelial colony-forming cells (ECFCs) emerge after 14–21 days of culture^14^. MACs are less proliferative and express high levels of CD14, MAC-1 and CD11c, which also mark monocyte/macrophages, but express low levels of endothelial marker VE-cadherin (CDH5) and stem-cell markers CD133 and c-Kit^15^. Contrastingly, ECFCs are highly proliferative and positive for CD34, CD105, CD146, CD31 and VE-cadherin^10^. Both MACs and ECFCs are present in humans, while only MACs are reported in mice. Though being extensively studied for more than two decades, the in vivo evidence of EPCs is still scarce.

Accumulating evidence suggests that a decline in the function and/or number of TSCs is responsible for the reduced regeneration capacity seen in aging and age-related diseases^16–22^. The function and/or number of HSCs and MSCs, in particular, decline with aging^23,24^. In a mouse model resembling Hutchinson–Gilford progeria syndrome (HGPS), which is predominantly caused by *LMNA* mutations, there is a prominent and premature depletion of MSCs, HSCs, hair follicle stem cells and muscle stem cells^25–28^. An age-related decline in the number and function of EPCs is also evidenced, which could be the main reason for the reduced vascular endothelium (VE)-repair capacity^9,29,30^. However, fundamental questions remain. It is still unclear whether TSC decline, depletion or malfunction is a cause, rather than a consequence, of aging, and whether replenishment with healthy and young TSCs could rejuvenate aged tissues/organs and promote longevity. Recently, we and others have shown that VE dysfunction causes systemic aging in mice^31–33^, raising the question of whether EPC decline, if any, causally accelerates aging.

In this Article, we aimed to clarify the origin and identity of potential EPCs and test the hypothesis that declines in EPCs drive aging. By lineage tracing with various genetic modifying strategies, single-cell transcriptomics and functional analyses, we revealed that CD133 labels potential EPCs in the BM, namely endothelial-like cells (ELCs). Both premature and physiological aging declined the function of CD133^+^ ELCs, and the replenishment with young and healthy ELCs rejuvenated aged blood vessels and promoted longevity in mice.

## Results

### BMNCs differentiate into ECs in distant organs

**Fig. 1:**
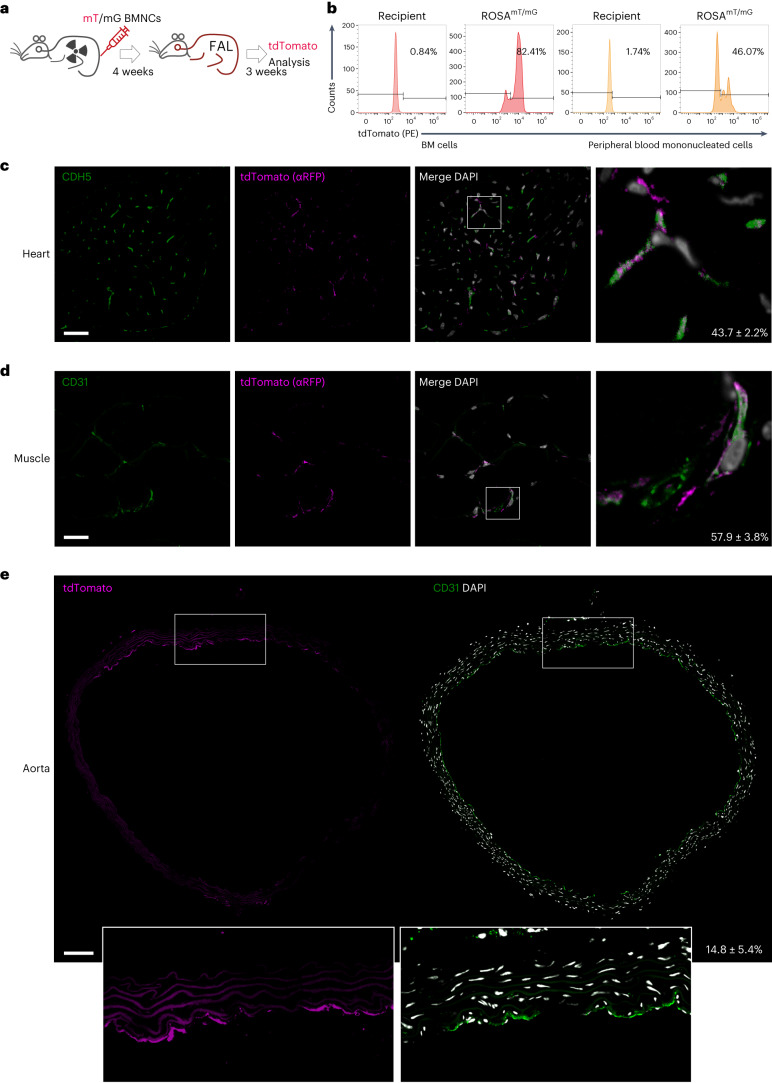
BMNCs differentiate into ECs in distant organs. **a**, Schematic of lineage-tracing strategy: recipient mice were irradiated with X-rays and transplanted with BMNCs from ROSA^mT/mG^ mice; 4 weeks after bone marrow transplantation (BMT), the recipient mice were subjected to unilateral FAL. Three weeks after FAL, the mice were killed for investigation: analysis of tdTomato^+^ endothelial cells (ECs). **b**, BM reconstitution rate determined by FACS of BM and peripheral blood cells. **c**, Representative fluorescence images showing donor-derived CDH5^+^ and tdTomato^+^ (anti-RFP) ECs in heart tissues. Scale bar, 20 µm. **d**, Representative fluorescence images showing donor-derived CD31^+^ and tdTomato^+^ (anti-RFP) ECs in muscle tissues. Scale bar, 20 µm. **e**, Representative fluorescence images showing donor-derived CD31^+^ and tdTomato^+^ ECs in adjacent aorta tissue sections. Scale bar, 100 µm. Five mice were analyzed. Data represent the mean ± s.e.m. Pseudocolor scheme used in immunofluorescent images: white represents the nucleus stain (DAPI), and magenta represents red signal (tdTomato live signal or labeled with Alexa Fluor 594-conjugated anti-RFP antibodies). Source Data

**Extended Data Fig. 1:**
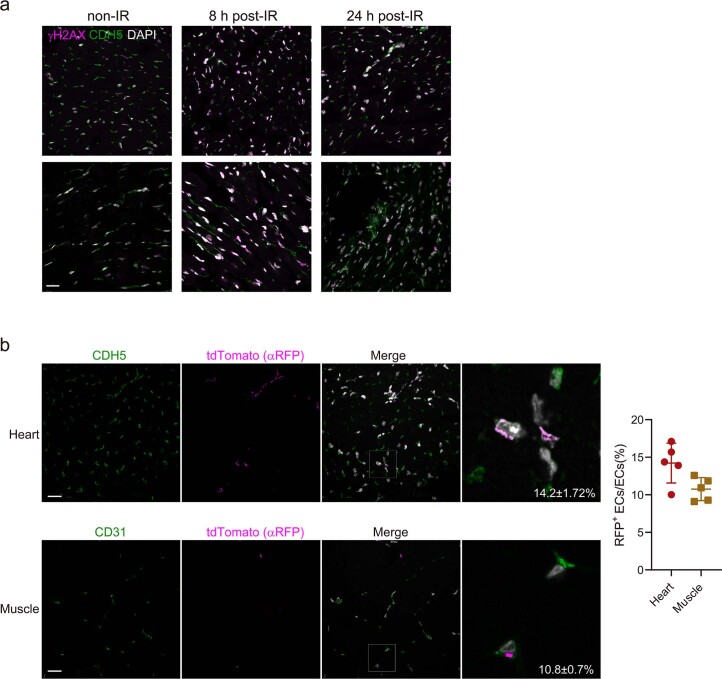
Irradiation induces DNA damage and endothelial cell turnover. (**a**) Representative fluorescence images with anti-γH2AX and anti-CDH5 co-staining in heart tissues isolated from mice after irradiation. Scale bar, 20 µm. 3 mice were analyzed. (**b**) Representative fluorescence images showing co-staining of anti-RFP (tdTomato) and anti-CDH5/CD31 in heart (upper) and muscle (lower) tissues isolated from mice BMTed with ROSA^mT/mG^ BMNCs. Scale bar, 20 µm. Quantification data represent the means ± s.e.m. 5 mice were analyzed. Source Data

**Extended Data Fig. 2:**
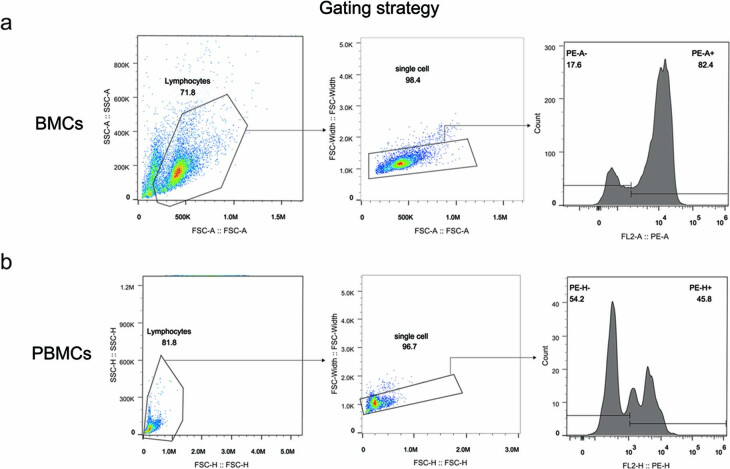
**FACS gating strategy.**

Given that endothelial and hematopoietic cells share the same origin, hemangioblasts^34^, we reasoned that EPCs might originate from the BM. To test this hypothesis, we employed a lineage-tracing strategy (Fig. 1a): The bone marrow mononuclear cells (BMNCs) from ROSA^mT/mG^ mice, which harbor a tdTomato and enhanced green fluorescent protein (EGFP) expression cassette, were transplanted into C57BL/6 recipient mice irradiated with a lethal dose of X-rays (9 Gy). X-ray irradiation introduced substantial amount of DNA damage, as indicated by γH2AX fluorescence staining (Extended Data Fig. 1a). The recipient mice were allowed to recover for 4 weeks and then subjected to unilateral femoral artery ligation (FAL) to stimulate neovascularization. After 3 weeks, the mice were killed for further investigation. The degree of BM repopulation was up to 80%, and that of the peripheral blood repopulation was close to 50% (Fig. 1b and Extended Data Fig. 2).

**Extended Data Fig. 3:**
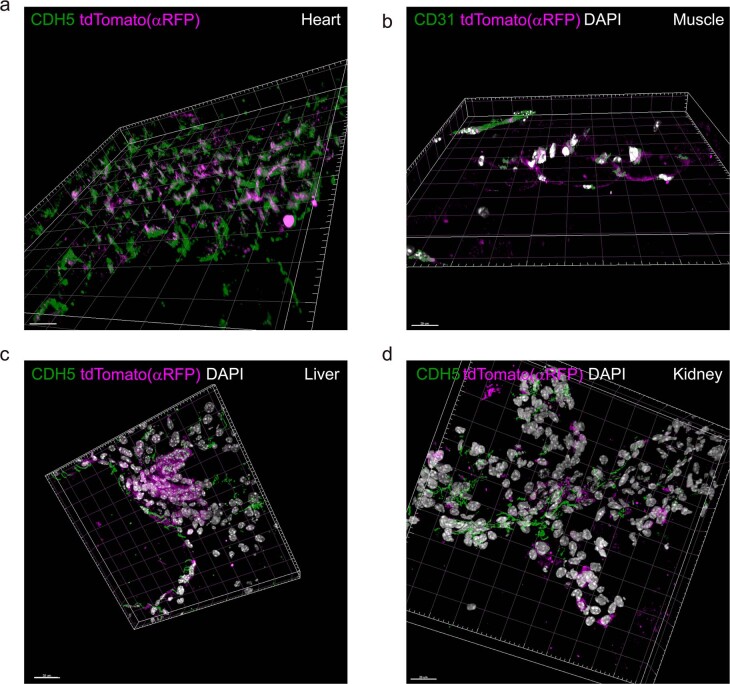
3D visualization of co-immunofluorescence-stained ECs. (**a**-**d**) 3D visualization of CDH5/RFP (tdTomato) co-staining in heart (a), liver (c), and kidney (**d**), and CD31/RFP co-staining in muscle (**b**). Scale bar, 20 µm. 3 mice were analyzed.

**Extended Data Fig. 4:**
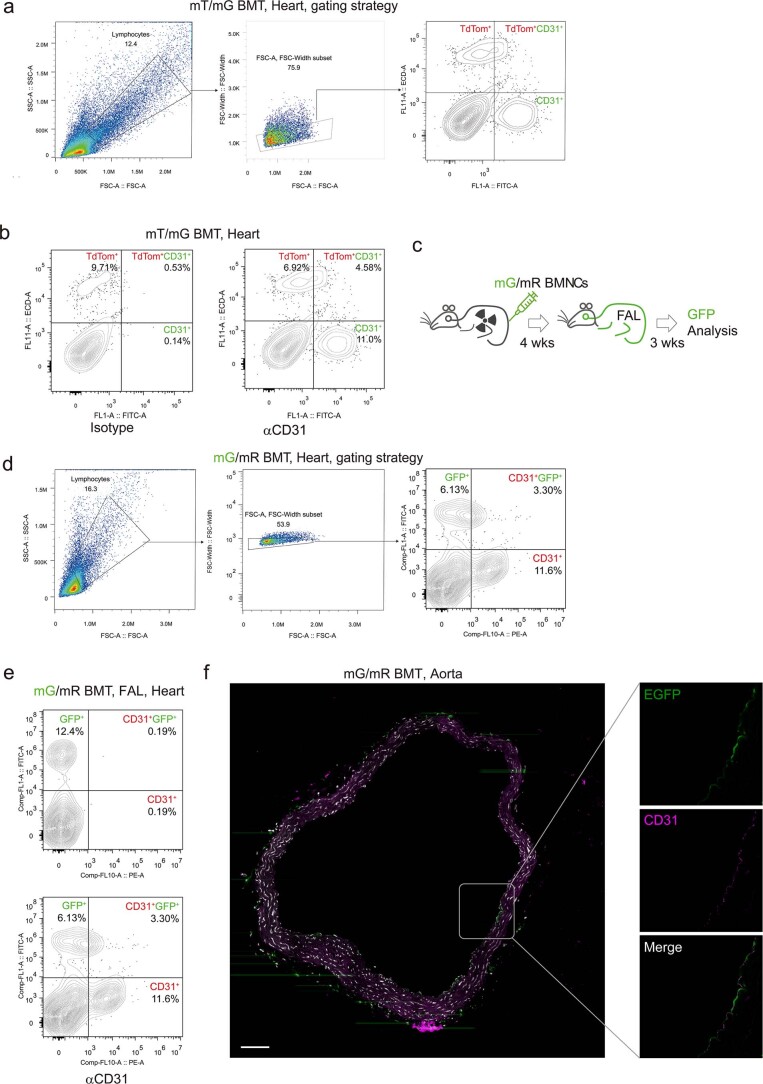
Single cell profiling in heart and aorta tissues. (**a**) FACS gating strategy. (**b**) FACS analysis of tdTomato (Tom)^+^, CD31^+^, and tdTomato (Tom)^+^CD31^+^ cells in heart tissues from ROSA^mT/mG^ BMTed mice. FITC, CD31^+^; ECD, tdTomato^+^. 5 mice were analyzed. (**c**) Schematic of lineage-tracing strategy: recipient mice were irradiated with a lethal dose of X-rays and transplanted with BMNCs from H11-mG/mR mice; 4 weeks after BMT, the recipient mice were subjected to unilateral FAL. Three weeks after FAL, the mice were sacrificed for investigation. (**d**) FACS gating strategy. (**e**) FACS analysis showing the proportion of donor-derived GFP^+^CD31^+^ ECs in heart tissue from H11-mG/mR BMNCs transplanted mice. FITC, GFP positive; PE, CD31 positive. (**f**) Representative fluorescence images showing donor-derived CD31^+^(red) GFP^+^ ECs in the aorta tissue sections.5 mice were analyzed.

We examined whether the donor-derived BMNCs could differentiate into ECs using immunofluorescent microscopic examination of anti-tdTomato and anti-CDH5 or anti-CD31 double staining. As shown, a substantial number of tdTomato^+^ cells were concomitantly positive for CDH5 or CD31 staining in heart and muscle tissues in bone-marrow-transplanted (BMTed) mice (Fig. 1c,d). Of note, irradiation enhanced the turnover rate of endothelial cells in heart and muscle tissues (Extended Data Fig. 1b). More interestingly, approximately 43.7 ± 2.2% of CDH5^+^ cells in the heart were positive for tdTomato, indicating their donor BMNC origin, and similarly 57.9 ± 3.8% of CD31^+^ cells in muscle tissues originated from donor BMNCs. Paralleled imaging of tdTomato live and anti-CD31 staining fluorescence signals in adjacent aorta tissue sections revealed that a large proportion of CD31^+^ ECs was derived from ROSA^mT/mG^ BMNCs (Fig. 1e). Conversely, donor-derived cells were barely detectable in the livers, lungs and kidneys (Supplementary Fig. 1). To exclude the possibility that the colocalized fluorescence signal was an artifact of image processing of two adjacent cells respectively positive for tdTomato or CD31, we performed multiple-layer image acquisition and 3D construction. The colocalized tdTomato (indicated by anti-red fluorescence protein (RFP) staining) and anti-CD31/CDH5 signals were observed at individual cell level of both heart and muscle tissues, but such colocalization was barely observed in the liver and kidney tissues (Extended Data Fig. 3 and Supplementary Videos 1–4). We also used flow cytometry to detect the donor-derived ECs in the heart tissues of BMTed mice at single-cell resolution. Approximately 30% of ECs were labeled with tdTomato (Extended Data Fig. 4a,b), indicating their BMNC origin.

To affirm that BM-derived EPCs can differentiate into ECs in distant organs, we used another independent lineage-tracing mouse model: H11-mG/mR mice, which harbor a CAG–LoxP–ZsGFP–Stop–LoxP–tdTomato cassette. BMNCs from H11-mG/mR mice were transplanted into C57BL/6 recipient mice irradiated with a lethal dose of X-rays. The recipient mice were allowed to recover for 4 weeks followed by unilateral FAL. After 3 weeks, mice were killed for investigation (Extended Data Fig. 4c). We found that approximately 22% of ECs in the heart tissues were labeled with green fluorescent protein (GFP) (Extended Data Fig. 4d,e). Anti-CD31 immunofluorescence staining in thoracic aorta sections revealed that a substantial number of ECs were differentiated from ZsGFP-labeled BMNCs (Extended Data Fig. 4f).

Next, we generated a recombinant adeno-associated virus serotype 1 (rAAV1) with a synthetic *ICAM2* promoter which drives vascular endothelial-specific expression of Cre. The rAAV1–ICAM2–cre particles were injected into the ROSA^mT/mG^ BMTed mice via tail vein with a dose of 1 × 10^11^ vector genomes/200 μl per mouse. After 4 weeks, mice were euthanized, and tissues were collected for FACS analysis (Supplementary Fig. 2a). If BMTed BMNCs can differentiate into ECs, tdTomato-labeled, GFP-labeled (under the action of Cre recombinase for long period of time) and both tdTomato- and GFP-labeled cells would be observed. The results showed that, of fluorescence-labeled cells in the heart tissues, about 21% expressed tdTomato only, labeling donor BMNCs, which had not differentiated into ECs, ~6% were positive for both tdTomato and GFP, representing the donor cells that were undergoing differentiation, and more than 70% cells were labeled with GFP only, indicating ECs differentiated from BMTed BMNCs (Supplementary Fig. 2b). In contrast, the GFP signal was barely detectable in the other tissues (Supplementary Fig. 2c). GFP^+^ cells were also detected in the heart tissues using immunofluorescence microscopy (Supplementary Fig. 2d)

**Extended Data Fig. 5:**
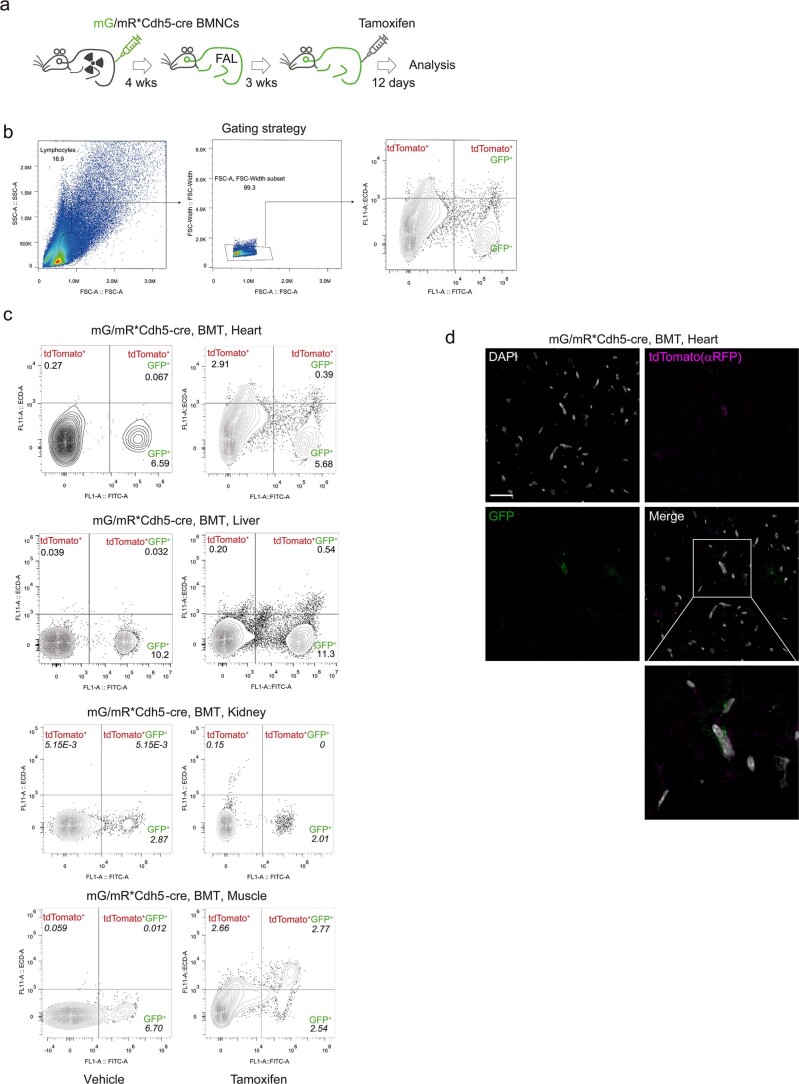
FACS and microscopy analysis of BMTed cells labeled by induced Cre expression. (**a**) Schematic of lineage-tracing strategy: recipient mice were irradiated with X-rays and transplanted with BMNCs from H11-mG/mR;Cdh5-cre/ERT mice; 4 weeks after BMT, the recipient mice were subjected to unilateral FAL. Three weeks after FAL, the mice were intraperitoneally injected with Tamoxifen for five days. Seven days later, mice were euthanized, and multiple tissues were collected for flow cytometry analysis. (**b**) FACS gating strategy. (**c**) FACS analysis of fluorescence cells in heart, liver, kidney and muscle tissues from H11-mG/mR;Cdh5-cre/ERT BMTed mice treated with or without Tamoxifen. FITC, GFP positive; ECD, tdTomato positive. (**d**) Representative fluorescence images showing donor derived tdTomato^+^GFP^+^ cells in heart tissues from H11-mG/mR;Cdh5- cre/ERT BMTed mice treated with Tamoxifen. 5 mice were analyzed.

Further, we crossed the H11-mG/mR mice with an inducible Cdh5–cre/ERT mouse line to generate mG/mR;Cdh5–cre mice, whereby the expression of Cre is driven by the promoter of endothelial-specific *Cdh5* gene in the presence of tamoxifen. BMNCs were collected from mG/mR;Cdh5–cre mice and transplanted into C57BL/6N mice irradiated with a lethal dose of X-rays. After 4 weeks’ recovery, recipient mice were subjected to unilateral FAL. Three weeks later, BMTed mice were intraperitoneally injected with tamoxifen (70 mg kg^−1^ per day) for 5 days. Seven days later, the mice were euthanized, and tissues were collected for flow cytometry analysis (Extended Data Fig. 5a). If the ZsGFP-labeled BMNCs can differentiate into ECs, under the action of Cre induced by tamoxifen, they would express tdTomato with or without GFP. Of all fluorescence-labeled cells in heart tissues, about 56% of them carried tdTomato only, representing ECs differentiated from BMTed BMNCs, and 15% carried both GFP and tdTomato, which, most likely, were differentiating or had just differentiated into ECs (Extended Data Fig. 5b,c). In contrast, tdTomato-positive cells were hardly detectable in liver, kidney and muscle tissues. We also prepared frozen sections and examined the cells expressing tdTomato using immunofluorescence microscopy (Extended Data Fig. 5d).

**Extended Data Fig. 6:**
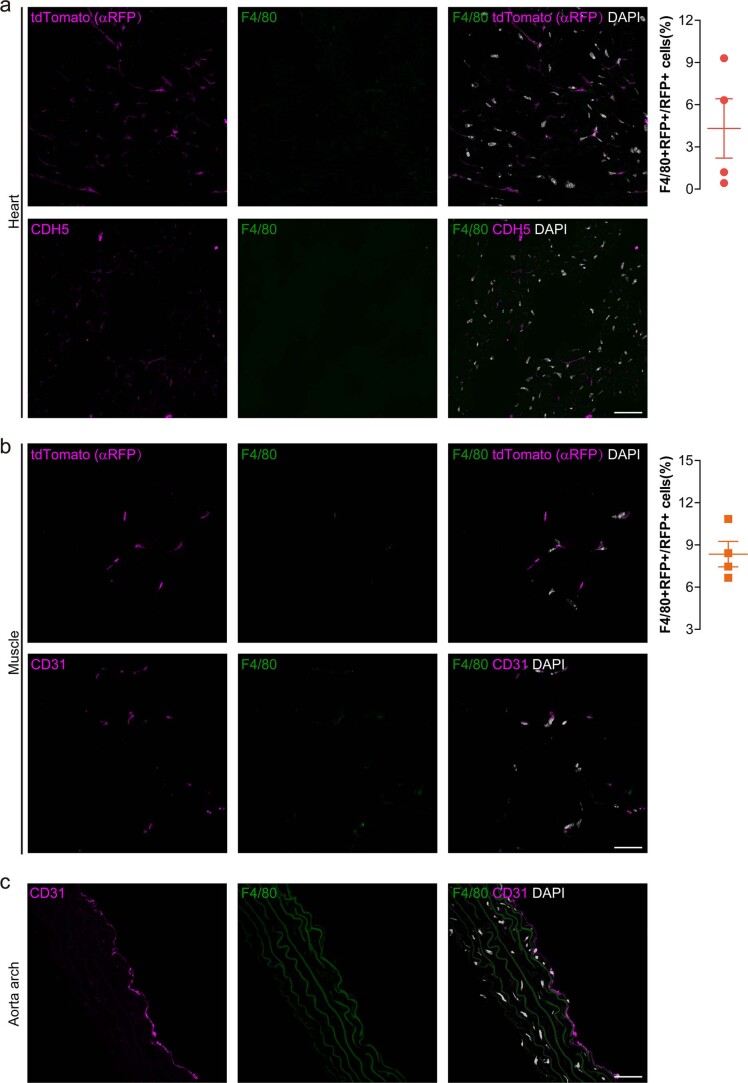
Donor-derived ECs seldomly originate from macrophages. (**a**) Representative fluorescence images showing co-staining of anti-RFP (tdTomato) and F4/80 (upper) and anti-CDH5 and F4/80 (lower) in heart tissue isolated from mice BMTed with rosa26-mT/mG BMNCs. The percent of F4/80+ cells in donor-derived RFP+ cells is shown (right). Scale bar, 20 µm. (**b**) Representative fluorescence images showing co-staining of anti-RFP (tdTomato) and F4/80 (upper) and anti-CD31 and F4/80 (lower) in muscle isolated from mice BMTed with ROSA^mT/mG^ BMNCs. The percent of F4/80+ cells in donor-derived RFP+ cells is shown (right). Scale bar, 20 µm. (**c**) Representative fluorescence images showing co-staining with anti-CD31 and F4/80 in aortic arch from mice BMTed with ROSA^mT/mG^ BMNCs. Note that F4/80+ cells were only detected in the aortic endothelium. Scale bar, 100 µm. Data represent the means ± s.e.m. 4 mice were examined. Source Data

In addition to direct differentiation, the fusion of local recipient ECs with donor cells, passive diffusion of fluorescence proteins to adjacent cells, and trans-differentiation of macrophages to ECs may cause the observed results. To exclude such possibilities, we first employed the Tie2–cre mouse line as recipients for BM reconstitution with ROSA^mT/mG^ BMNCs (Supplementary Fig. 3a). If fusion had taken place, Cre recombinase originating from the recipient ECs would have worked with the ROSA^mT/mG^ locus in the donor BMNCs and activated EGFP expression; if passive diffusion of tdTomato had occurred, it would be detected in Cre-positive ECs. While tdTomato^+^ ECs were frequently observed in heart, muscle and aorta tissues, EGFP^+^ ECs were scarce (Supplementary Fig. 3b–d). Notably, tdTomato^+^ cells lacked Cre staining (Supplementary Fig. 3d). Lastly, we performed F4/80 staining to check whether the observed tdTomato^+^ ECs were macrophages. Though we observed F4/80 and tdTomato double-positive ECs, they were at a low frequency (Extended Data Fig. 6). Together, all these data suggest that potential EPCs reside in the BM and can differentiate into ECs in distant organs of the BMTed model to maintain endothelial homeostasis.

### CD133 marks potential EPCs in mouse BM

**Fig. 2:**
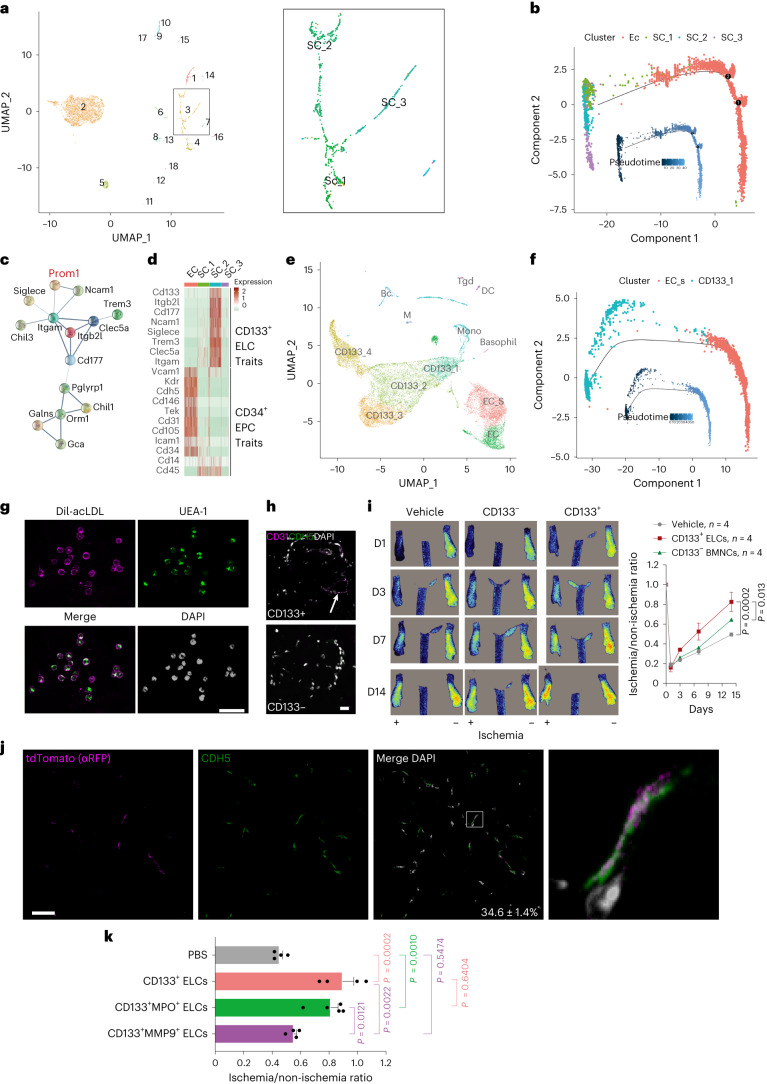
CD133-marked ELCs implicate angiogenic potential. **a**, Pseudo-time analysis of single-cell transcriptomics of whole mouse BMNCs and CDH5^+^ BM niche ECs. Numbers represent proximity on pseudo-timeline rather than chronological order. Note that ECs (cluster 2) are more closely related to cluster 3, which is further divided into subclusters SC_1, SC_2 and SC_3. **b**, Pseudo-timeline analysis shows evolutionary trends for clusters EC, SC_1, SC_2 and SC_3, of which clusters EC and SC_2 are more closely related. **c**, Most enriched pathways of genes with unique expression in cluster SC_2 relative to other clusters of BMNCs (Extended Data Fig. 7d). Note the enriched cell surface proteins. **d**, Heatmap analysis of genes revealed in **c** and genes used to identify EPCs in the literature, namely CD34^+^ EPC traits across SC_1, 2 and 3, and ECs. **e**, Uniform Manifold Approximation and Projection (UMAP) visualization of single-cell transcriptomes of CD133^+^ ELCs, whole mouse BMNCs and CDH5^+^ BM niche ECs. CD133^+^ ELCs were clustered into CD133_1‒4. Three mice were included for analyses. **f**, Pseudo-time analysis of EC subcluster EC_S and ELC subcluster CD133_1. **g**, CD133^+^ ELCs labeled with the Dil-conjugated acetylated low-density lipoproteins (Dil-acLDL) and UEA-1. Three mice were examined. Scale bar, 50 µm. **h**, Ex vivo Matrigel plug assay showing tubular-like structure with CD31^+^Cdh5^+^ cells, which was not observed in CD133^‒^ BMNC plugs. Scale bar, 20 µm. The arrow indicates a tubular-like structure. All images are representative for three animals in each group and two implants per mouse. **i**, Representative microcirculation images (left) and quantification of blood flow recovery (right) following hind limb ischemia in mice treated with CD133^+^ ELCs, CD133^‒^ BMNCs or without ELCs/BMNCs. *n* = 4 mice per group. D, experimental day. *P* value calculated by two-way ANOVA. **j**, Representative fluorescence images showing CDH5^+^ ECs and tdTomato^+^ (anti-RFP) donor-derived cells in muscle tissue. Four mice were examined. Scale bar, 20 µm. **k**, Blood flow recovery rate on day 14 after the hind limb ischemia in mice treated with CD133^+^, CD133^+^MPO^+^, CD133^+^MMP9^+^ BMNCs or PBS (*n* = 4, 4, 5 and 4, respectively). *P* value calculated by Tukey’s multiple comparisons one-sided one-way ANOVA test. Data represent the mean ± s.e.m. Pseudocolor scheme used in immunofluorescent images: white represents the nucleus stain (DAPI), and magenta represents red signal (tdTomato (RFP)/CD31 labeled with Alexa Fluor 594-conjugated antibodies or Dil-acLDL). Source Data

**Extended Data Fig. 7:**
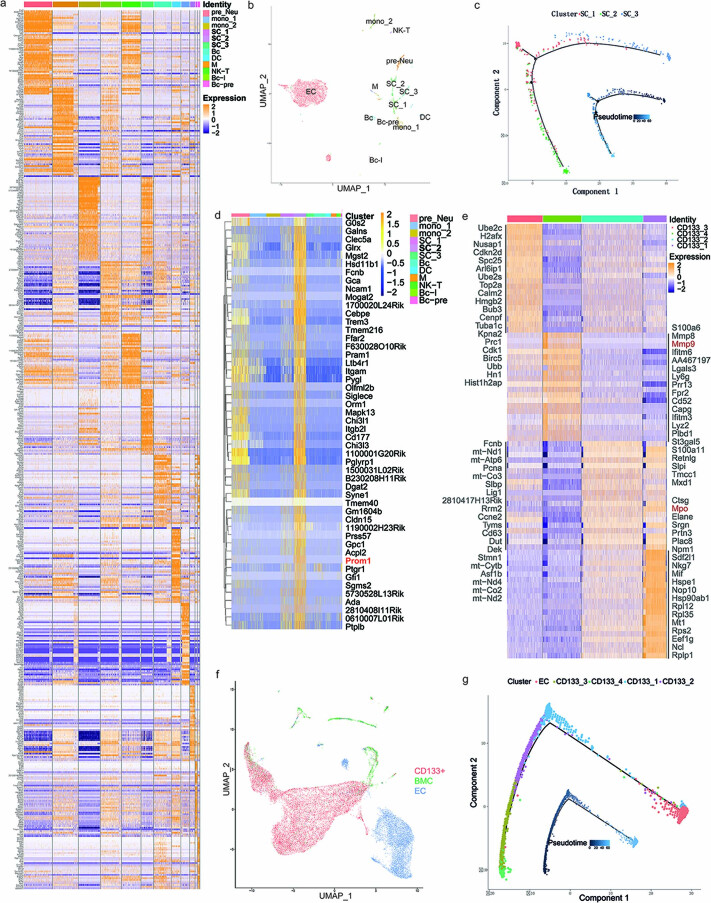
CD133 marks a unique cell population in BMNCs. (**a**) Identification of whole-mouse BMNCs and expression of top-100 marker genes in different cell types (clusters). The marker genes are also listed in Supplementary Table 1. (**b**) Cell types (clusters) of whole-mouse BMNCs and CDH5^+^ BM niche ECs, related to Fig. 2a. (**c**) Pseudo-time analysis of subclusters SC_1, SC_2, and SC_3. (**d**) Heatmap showing top-50 marker genes for subcluster SC_2. (**e**) Heatmap showing top marker genes for clusters CD133_1‒4. (**f**) UMAP visualization of cell source: whole-mouse BMNCs, CDH5^+^ BM niche ECs, and CD133^+^ ELCs. (**g**) Pseudo-temporal relationship between CDH5^+^ BM niche ECs and CD133^+^ ELCs (clusters CD133_1‒4).

Having shown the EC differentiation potential of BMNCs, we sought to further enrich the EPC subpopulation. We reasoned that EPCs should be more closely related to ECs in the BM. To test the hypothesis, we first employed the single-cell RNA sequencing data from two studies describing whole mouse BMNCs and BM niche CDH5^+^ cells, named ECs^35,36^. BMNCs were re-clustered according to marker-gene expression (Extended Data Fig. 7a and Supplementary Table 1). The two datasets were integrated after batch effects were removed. Interestingly, a pseudo-time analysis of all cells revealed that BMNC clusters SC_1, SC_2 and SC_3, which were predicted to have stem cell/progenitor properties, were closely related to ECs (Fig. 2a and Extended Data Fig. 7b). Further pseudo-time analyses revealed that SC_2 had more progenitor properties and was predicted to differentiate into SC_1 or SC_3 (Extended Data Fig. 7c), and SC_2 most likely represented EPCs that reside upstream of ECs (Fig. 2b).

We next examined the transcriptomes of the three clusters and other populations in the BM to identify putative molecular signature(s) for cluster SC_2. Interestingly, a group of cell-membrane-associated components marked the cluster SC_2, of which CD133 encoded by the *Prom1* gene was the most prominent (Fig. 2c and Extended Data Fig. 7d). We referred to this putative marker set as CD133^+^ cell traits and compared its expression with reported EPC markers: CD45, CD14 and CD31 for MACs; CD105, CD14 and CD31 for ECFCs; VCAM1, ICAM1 and KDR for ECs; and CD34, collectively named CD34^+^ EPC traits. Along with the pseudo-timeline (Fig. 2b), the CD133^+^ cell traits were predominantly expressed in SC_2, sharply declined in SC_1, and almost disappeared in ECs (Fig. 2d). Of CD34^+^ EPC traits, CD14 expression was highest in SC_2, then SC_1, and lowest in ECs. All the others were highly expressed in ECs, with certain extent expression of CD34 and ICAM1 in SC_1 and barely any in SC_2. We reasoned that CD133 might label the early stages of putative EPCs, referred to as CD133^+^ ELCs.

We thus applied fluorescence-activated cell sorting (FACS) to enrich CD133^+^ BMNCs from three C57BL/6N mice. To experimentally validate the properties of the CD133^+^ ELCs, we did 10x Genomics single-cell RNA sequencing, and after quality filtering, 10,869 cells were recovered. By employing the *k* means clustering algorithm, the CD133^+^ ELCs were further clustered into four groups: CD133_1, CD133_2, CD133_3 and CD133_4 (Fig. 2e and Extended Data Fig. 7e,f). Interestingly, while *Prom1* was ubiquitously expressed in all four subclusters, *Mpo* gene encoding myeloperoxidase was mainly expressed in cluster CD133_1 and *Mmp9* was mainly expressed in cluster CD133_4 (Fig. 2e and Supplementary Fig. 4). In the pseudo-time axis, cluster CD133_1 was more strongly associated with ECs (Fig. 2f and Extended Data Fig. 7g).

We further investigated the functional properties of CD133^+^ ELCs. EPCs are known to take up low-density lipoproteins (LDLs) and *Ulex europaeus* agglutinin 1 (UEA-1)^11^. More than 95% of freshly FACS-isolated CD133^+^ ELCs were positive for Dil-acLDL and UEA-1 (Fig. 2g), indicating their endothelial potential. To study the potential of CD133^+^ ELCs in neovascularization, we conducted ex vivo Matrigel plug assay, and CD133^+^ ELC plugs showed tubular-like structures with CD31^+^Cdh5^+^ cells, which were not observed in CD133^‒^ BMNC plugs (Fig. 2h). We then enriched the CD133^+^ ELCs and CD133^‒^ BMNCs from ROSA^mT/mG^ mice using magnet-activated cell sorting (MACS) and intravenously injected them into mice given FAL via the tail vein. Dynamic microcirculation imaging system analysis showed the blood flow recovery rate on day 14 was significantly enhanced from 50% to almost 85% after CD133^+^ ELC supplementation, but that was only 65% after CD133^‒^ BMNC treatment (Fig. 2i). Donor-derived tdTomato-expressing ECs were frequently observed at injury sites, and about 34.6 ± 1.4% CD31^+^ cells were positive for tdTomato (Fig. 2j). Of particular note, mice treated with CD133^+^ ELCs and CD133^+^MPO^+^ ELCs that represent cluster CD133_1 showed comparable blood flow recovery rate on day 14 after FAL (Fig. 2k). The treatment with CD133^+^MMP9^+^ ELCs that represent CD133_4 barely restored the blood flow. Collectively, these data suggest that the CD133^+^ ELCs in the BM, mainly represented by a CD133^+^MPO^+^ subcluster, promote neovascularization.

### CD133^+^ ELCs ameliorate features of premature aging in mice

**Fig. 3:**
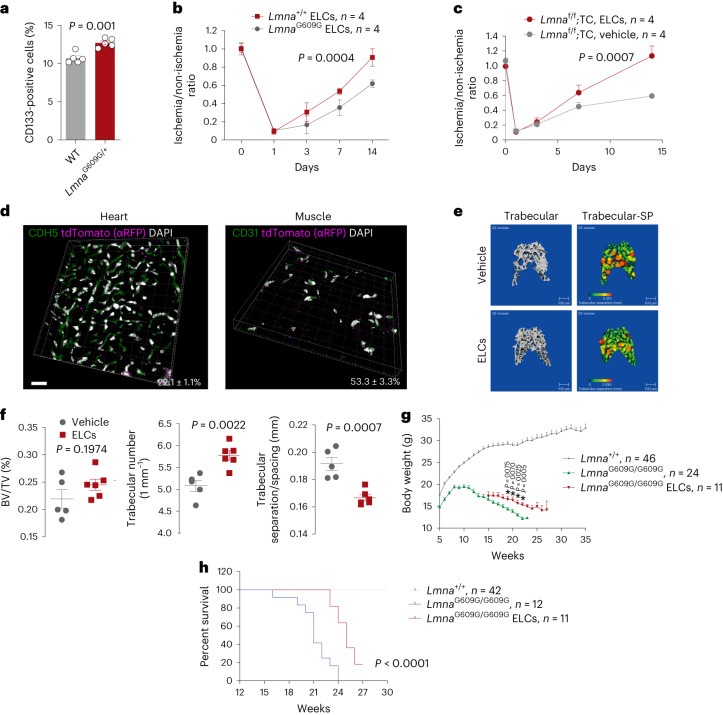
Rejuvenation of CD133^+^ ELCs ameliorates aging and extends lifespan of progeria mice. **a**, Percentage of CD133^+^ ELCs in total BMNCs isolated from *Lmna*^G609G/+^ mice (*n* = 5) and WT mice (*n* = 5). *P* value calculated by one-sided Student’s *t*-test. **b**, Blood flow recovery rate of *Lmna*^+/+^ CD133^+^ ELC- and *Lmna*^G609G/G609G^ CD133^+^ ELC-treated WT C57BL/N mice given FAL surgery. *P* value calculated by two-way ANOVA. **c**, Blood flow recovery rate of *Lmna*^+/+^ CD133^+^ ELC- and vehicle-treated *Lmna*^f/f^;TC mice given FAL surgery. *P* value calculated by two-way ANOVA. **d**, Representative fluorescence images showing CDH5^+^ ECs and tdTomato (RFP)^+^ donor-derived cells in heart tissues, and CD31^+^ ECs and RFP^+^ donor-derived cells in muscle tissues. Three mice were examined. **e**,**f**, Representative 3D reconstruction images of the trabecular bone determined by micro-CT (**e**), and quantitative analysis (**f**) of BV/TV, trabecular number and trabecular SP of *Lmna*^G609G/G609G^ mice treated with WT ELCs (*n* = 5) or vehicle (*n* = 8). *P* value calculated by one-sided Student’s *t*-test. **g**, Body weight of ELC-treated and untreated *Lmna*^G609G/G609G^ and *Lmna*^+/+^ mice. *P* value calculated by one-sided multiple *t*-test. **h**, Lifespan of ELC-treated and untreated *Lmna*^G609G/G609G^ and *Lmna*^+/+^ mice. Scale bar, 20 µm. Data represent the mean ± s.e.m. log-rank (Mantel–Cox) test was used for survival analyses. Pseudocolor scheme used in immunofluorescent images: white represents the nucleus stain (DAPI), and magenta represents red signal (tdTomato (RFP) labeled with Alexa Fluor 594-conjugated antibodies). Source Data

**Extended Data Fig. 8:**
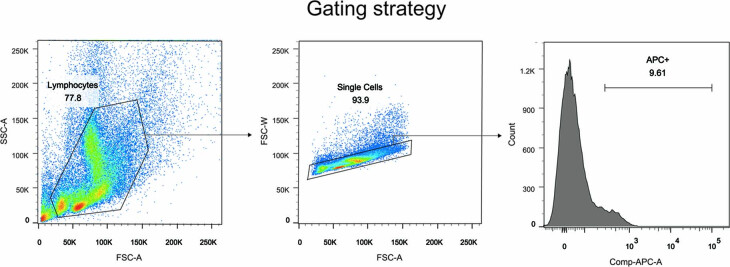
**FACS gating strategy**.

Endothelial dysfunction accelerates aging and shortens the lifespan of a mouse model resembling HGPS: *Lmna*^f/f^;TC mice^33^. We hypothesized that endothelial dysfunction is attributable to the defects in EPCs, and the replenishment of EPCs might extend the model’s lifespan. To test these hypotheses, we first analyzed the number of CD133^+^ ELCs in *Lmna*^G609G/+^ and wild-type (WT) control mice by FACS. The number of CD133^+^ ELCs was significantly increased in *Lmna*^G609G/+^ mice compared with that in control mice (Fig. 3a and Extended Data Fig. 8). Next, we enriched the CD133^+^ ELCs and studied their function in blood vessel regeneration in a FAL model. The neovascularization capacity of CD133^+^ ELCs from the global HGPS model, *Lmna*^G609G/G609G^ mice^33^, was compromised by roughly 30% compared with the controls (Fig. 3b), suggesting there was a functional decline in *Lmna*^G609G/G609G^ CD133^+^ ELCs. We then explored if replenishing CD133^+^ ELCs could rescue neovascularization in *Lmna*^f/f^;TC mice. Indeed, on-site injection of CD133^+^ ELCs isolated from ROSA^mT/mG^ mice completely restored the defective neovascularization in *Lmna*^f/f^;TC mice (Fig. 3c).

*Lmna*^G609G/G609G^ mice start to develop features of aging at 2 months of age and die 4‒6 months after birth^37^. We next questioned whether young and healthy CD133^+^ ELCs can rejuvenate aged blood vessels and ameliorate premature aging in progeria mice. To test these hypotheses, we enriched CD133^+^ ELCs from 3-month-old and healthy ROSA^mT/mG^ mice by MACS. Then, 1 × 10^6^ CD133^+^ ELCs were injected into *Lmna*^G609G/G609G^ mice via the tail vein, starting from 3 months of age (before the earliest death event) and every week for 1 month. Donor-derived tdTomato^+^ ECs were detected in the heart and muscle by fluorescence microscopy but were not seen in the aorta (Fig. 3d). More importantly, the trabecular number was significantly increased and trabecular separation/spacing was significantly decreased following the EPC replenishment in *Lmna*^G609G/G609G^ mice (Fig. 3e,f). Premature body weight loss was also attenuated by EPC therapy (Fig. 3g). Most importantly, the median lifespan of progeria mice was extended by almost 20% (from 21 to 25 weeks) (Fig. 3h). Together, these data suggest that CD133^+^ ELCs promote neovascularization and extend the lifespan of the premature aging murine model.

### CD133^+^ ELCs rejuvenate aged blood vessels and promote longevity

**Fig. 4:**
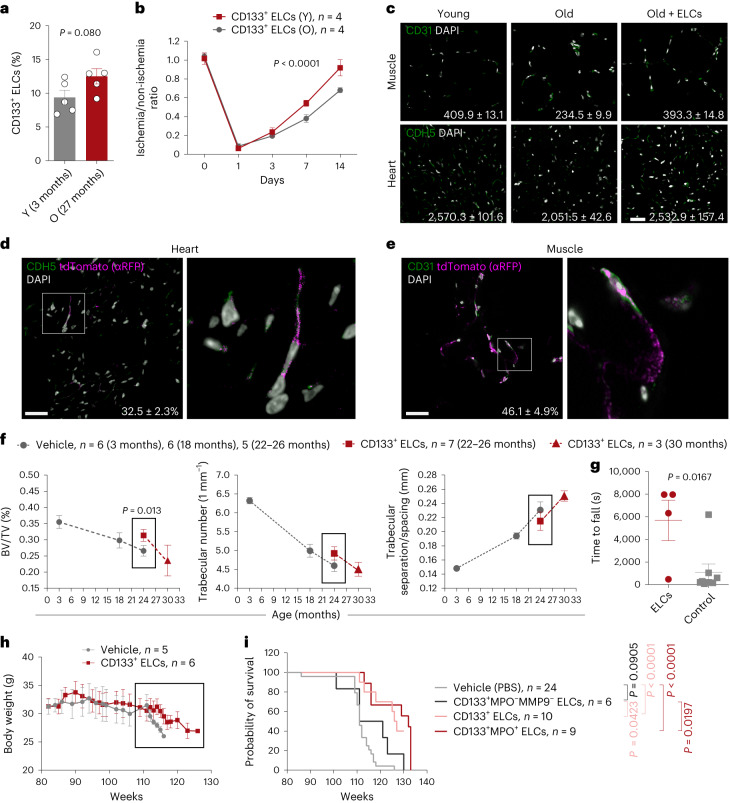
CD133^+^ ELC rejuvenation ameliorates aging and extends lifespan in physiologically aged mice. **a**, Percentage of CD133^+^ ELCs in total BMNCs isolated from young (3-month-old, Y) and old (27-month-old, O) mice (*n* = 5). *P* value calculated by one-sided Student’s *t*-test. **b**, Blood flow recovery rate of young (Y, 3-month-old) and old (O, 27-month-old) CD133^+^ ELC-treated WT C57BL/N mice given FAL surgery. *P* value calculated by two-way ANOVA. **c**, Representative fluorescence images showing CDH5^+^ ECs (heart) and CD31^+^ ECs (muscle) in young (4-month-old, *n* = 3) and old (26-month-old, *n* = 3) mice treated or untreated (*n* = 3) with ELCs isolated from young (3-month-old) mice. Capillary density (capillaries mm^−^^2^) is shown. **d**, Representative images showing CD31^+^ ECs and tdTomato (RFP)^+^ donor-derived cells in heart tissues. Three mice were examined. **e**, Representative images showing CD31^+^ ECs and tdTomato (RFP)^+^ donor-derived cells in muscle tissues. Three mice were examined. **f**, Micro-CT data of femurs isolated from mice at various ages treated with CD133^+^ ELCs from young mice or vehicle. *P* value calculated by one-sided Student’s *t*-test. **g**, Endurance of old mice (18 months old) treated with CD133^+^ ELCs isolated from young mice (*n* = 4) or vehicle (*n* = 6). *P* value calculated by one-sided Student’s *t*-test. **h**, Body weight of female mice treated with or without CD133^+^ ELCs. Open square indicates periods of body weight change between the two groups. **i**, Lifespan of CD133^+^ ELCs and subcluster-treated and untreated mice. Scale bars, 20 µm. Data represent the mean ± s.e.m. log-rank (Mantel–Cox) test was used for survival analyses. Pseudocolor scheme used in immunofluorescent images: white represents the nucleus stain (DAPI), and magenta represents red signal (tdTomato (RFP) labeled with Alexa Fluor 594-conjugated antibodies). Source Data

Vascular aging/dysfunction is a hallmark of aging; however, its causal roles still lack experimental support. Interestingly, while the number of CD133^+^ ELCs was not much changed in the older mice (27 months old, O) compared with the young mice (3 months old, Y) (Fig. 4a), the neovascularization capacity of ELCs (O) was significantly jeopardized compared with that of ELCs (Y) (Fig. 4b). The number of CD31^+^ ECs was substantially decreased in the hearts and muscles of old compared with young mice (Fig. 4c). We then investigated whether replenishing CD133^+^ ELCs enhances neovascularization capacity. To that end, 1 × 10^6^ ELCs enriched from 3-month-old ROSA^mT/mG^ mice were injected via the tail vein into 18-month-old mice every other week. Eight months after ELC replenishment, the capillary density (CD31^+^ ECs) of the gastrocnemius muscle had significantly increased, from 234.5 ± 9.9 mm^−^^2^ to 393.3 ± 14.8 mm^−^^2^ (*P* < 0.05), the latter of which is comparable to that of young mice (3 months old, 409.9 ± 13.1 mm^−^^2^). Correspondingly, the capillary density (CDH5^+^ ECs) in the heart also significantly increased after ELC treatment, from 2,051.5 ± 42.6 mm^−^^2^ to 2,532.9 ± 157.4 mm^−^^2^ (*P* < 0.05), which is again similar to that of young mice (3 months old, 2,570.3 ± 101.6 mm^−^^2^). Of particular interest, we consistently observed tdTomato^+^ cells in the heart and muscle tissues 8 months after the first injection of ELCs (Fig. 4d,e, Supplementary Fig. 5 and Supplementary Videos 5 and 6).

We next analyzed overall aging features in old ELC-treated mice. The bone volume (BV) to tissue volume (TV) ratio and the trabecular number progressively declined, whereas trabecular separation/spacing increased with aging (Fig. 4f). ELC therapy attenuated this exacerbation of bone loss, even reaching significance for BV/TV (%). ELC therapy also increased the running endurance (Fig. 4g). Furthermore, there was no change in body weight until the later stages of aging, when the untreated mice started to die (Fig. 4h). Most importantly, the median lifespan was extended by up to 10% from 115 to 126 weeks after CD133^+^ ELC replenishment. Further, we tested the lifespan-extending effect of the subclusters CD133^+^MPO^+^ and CD133^+^MPO^−^MMP9^−^ ELCs. Markedly, administration of CD133^+^MPO^+^ ELCs also extended the lifespan to a similar extent as the CD133^+^ ELCs did. In contrast, the replenishment of CD133^+^MPO^−^MMP9^−^ subcluster only slightly increased the lifespan but did not reach significance (Fig. 4i). Together, the data suggest that CD133^+^ ELCs, particularly the CD133^+^MPO^+^ subcluster, can rejuvenate aged blood vessels and promote longevity in mice.

### FDPS underlies ELC aging

**Fig. 5:**
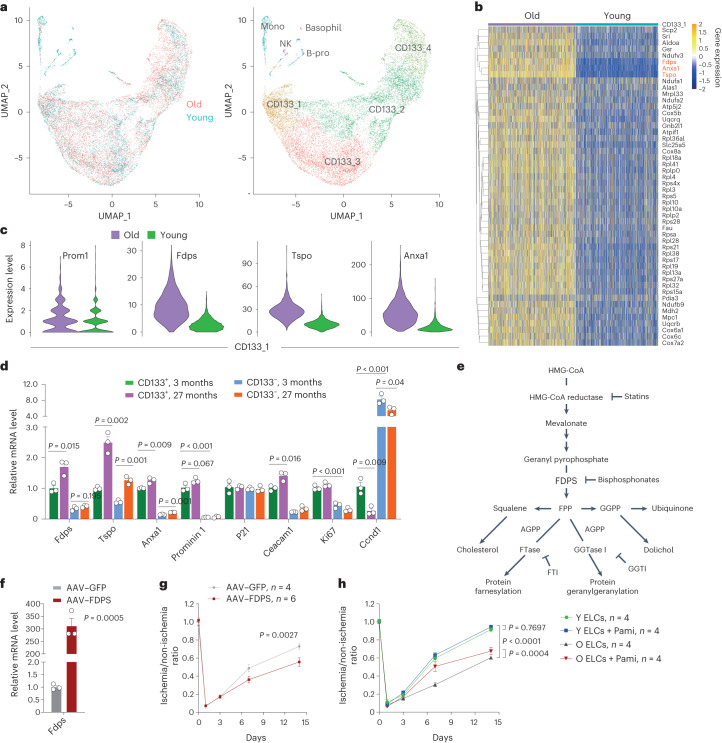
FDPS upregulation compromises ELC function. **a**, UMAP visualization of single-cell transcriptomes of CD133^+^ ELCs enriched from young (3-month-old, *n* = 3) and old (18.5-month-old, *n* = 3) mice, showing cell attribution (left) and identification of cells (right). **b**, Differential expression of ribosome- and oxidative phosphorylation-related genes in cluster CD133_1 between young and old mice. Note that *Fdps* was one of the three most significantly changed genes. **c**, Violin plot showing transcriptomic levels of genes *Prom1*, *Fdps*, *Tspo* and *Anxa1* in cluster CD133_1 from young and old mice. **d**, qPCR analysis of *Prom1*, *Fdps*, *Tspo* and *Anxa1* transcript levels in CD133^+^ ELCs and CD133^‒^ BMNCs from young (*n* = 3) and old mice (*n* = 3). The levels of *P21*, *Ceacam1*, *Ki67* and *Ccnd1* were included as control. *P* value calculated by one-sided Student’s *t*-test. **e**, FDPS-centered isoprenoid biosynthesis pathway. Note that PAM belongs to the bisphosphonates, which inhibit FDPS activity. **f**,**g**, Overexpression of *Fdps* via rAAVDJ in young CD133^+^ ELCs compromised their neovasculogenic capacity, as determined by FAL experiment (**f**, *n* = 3 mice per group; **g**, *n* = 4–6 mice per group). *P* value calculated by two-way ANOVA. **h**, Blood flow recovery following hind limb ischemia in mice treated with young or old ELCs and with or without PAM. *P* value calculated by two-way ANOVA. Data represent the mean ± s.e.m. Source Data

**Extended Data Fig. 9:**
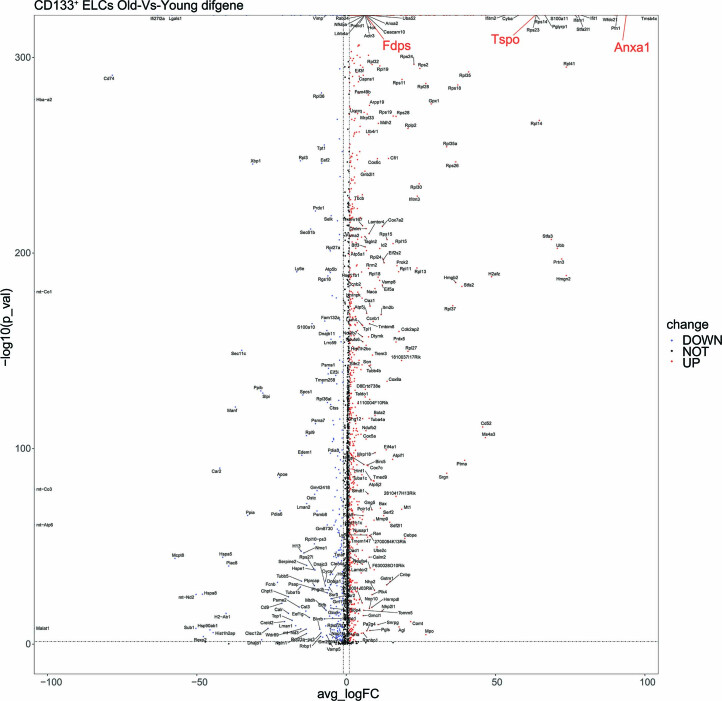
Differential gene expression between young and old CD133 + ELCs. Volcano map showing differential gene expression between CD133^+^ ELCs isolated from young and old mice. FC, fold change of gene expression; p_val, p-value. Differential expression was analyzed by non-parametric Wilcoxon-rank sum test.

**Extended Data Fig. 10:**
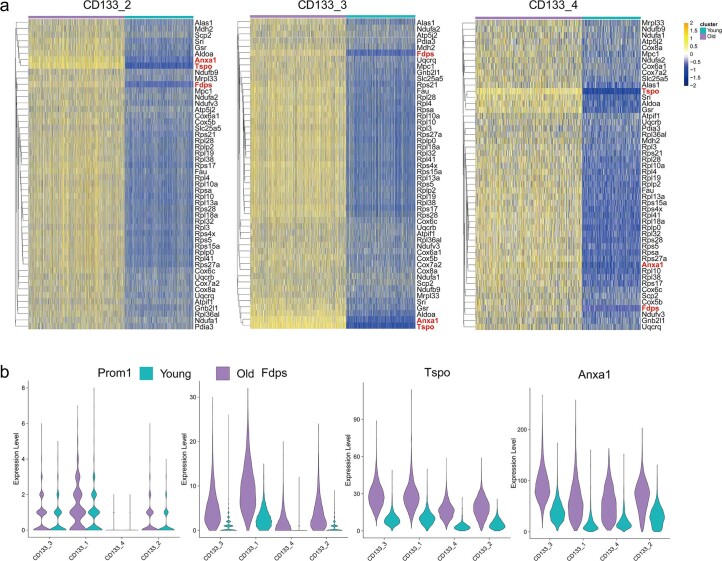
Differentially expressed genes between young and old CD133 + ELCs. (**a**) Differentially expressed genes in ribosome and oxidative phosphorylation pathways in clusters CD133_2–4 between young and old mice. Note that *Fdps*, *Tspo*, and *Anxa1* expression showed the greatest differences. (**b**) Violin plot showing transcriptomic levels of genes *Prom1*, *Fdps*, *Tspo*, and *Anxa1* in four clusters CD133_1–4.

To understand potential mechanisms that drive ELC aging and functional decline, we obtained and compared the single-cell transcriptomes of FACS-enriched CD133^+^ ELCs from young (3-month-old) and old (18.5-month-old) mice. We recovered 8,362 cells from young mice and 10,597 from old mice (Fig. 5a), of which approximately 454 genes were significantly upregulated, and 90 genes were significantly downregulated in ELCs isolated from old mice (Extended Data Fig. 9). Further functional enrichment analysis revealed two pathways that were significantly enriched in old CD133^+^ ELCs: ribosome biogenesis and oxidative phosphorylation (Supplementary Fig. 6a,b). The top-200 upregulated genes in the two pathways were selected for protein–protein interaction network analysis and gene expression comparison in the four clusters, CD133_1, CD133_2, Cd133_3 and CD133_4, between young and old mice. Very interestingly, three genes, *Fdps*, *Anxa1* and *Tspo*, were significantly and consistently upregulated in all four clusters of old ELCs (Fig. 5b,c and Extended Data Fig. 10a,b), and this was validated by quantitative polymerase chain reaction (PCR) with reverse transcription: *Fdps*, *Anxa1* and *Tspo* were all highly expressed in CD133^+^ cells (Fig. 5d). While expression levels of *Anxa1* and *Tspo* were significantly enhanced in both old CD133^+^ ELCs and CD133^−^ BMNCs, the upregulated expression of *Fdps* in old mice seemed more specific to CD133^+^ ELCs. Thus, we focused on *Fdps* for further mechanistic investigation. Of note, the CD133^+^ ELCs were more proliferative than CD133^−^ cells, as *Ki67* level was much higher in CD133^+^ ELCs than CD133^−^ BMNCs, while that of *Ccnd1* was much higher in CD133^−^ than CD133^+^ cells.

*Fdps* encodes farnesyl diphosphate synthase (FDPS), a key enzyme in isoprenoid biosynthesis that catalyzes the formation of farnesyl diphosphate^38^. Farnesyl diphosphate is a precursor for sterol, dolichol, carotenoid and ubiquinone and is also a substrate for protein farnesylation and geranylgeranylation (Fig. 5e). The diphosphonate derivative pamidronate (PAM) inhibits FDPS activity and is used clinically for the treatment of osteoporosis^39^. To determine if elevated *Fdps* expression underlies the functional decline of ELCs in old mice, we generated a recombinant AAV serotype DJ (rAAVDJ) vector with an *Fdps*-expressing cassette driven by a ubiquitous CMV promoter. The overexpression of *Fdps* was observed in CD133^+^ ELCs (Fig. 5f). In the FAL model, *Fdps* overexpression significantly compromised the new-blood-vessel-forming ability of ELCs derived from young mice (Fig. 5g). In contrast, incubation with PAM significantly improved the new-blood-vessel-forming ability of ELCs of old mice, whereas the combined treatment with PAM and young ELCs showed a minimal additive effect (Fig. 5h). Thus, an increase in *Fdps* compromises ELC function during aging.

### PAM treatment extends lifespan in mice

**Fig. 6:**
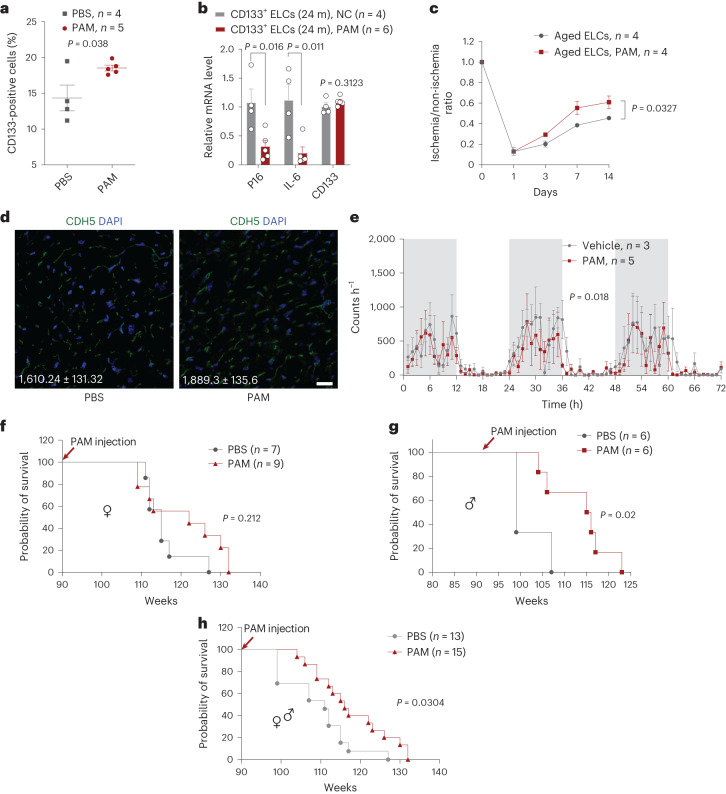
PAM ameliorates aging and extends lifespan in physiologically aged mice. **a**, Percentage of CD133^+^ ELCs in total BMNCs isolated from PAM-treated old mice and control mice (27-month-old, *n* = 5 and 4, respectively). *P* value calculated by one-sided Student’s *t*-test. **b**, qPCR analysis of *p16*, *Il-6* transcript levels in CD133^+^ ELCs from PAM-treated old mice and NC (PBS treatment) mice (24-month-old, *n* = 6 and 4, respectively). *P* value calculated by one-sided Student’s *t*-test. **c**, Blood flow recovery rate of FAL model treated with CD133^+^ ELCs isolated from the PAM-treated old mice or control mice (26-month-old). *P* value calculated by two-way ANOVA. **d**, Representative fluorescence images showing CDH5^+^ ECs in heart tissues of old (26-month-old, *n* = 4) mice treated or untreated (*n* = 4) with PAM. Capillary density (capillaries mm^−^^2^) is shown. **e**, Running activity of old mice treated with PAM or vehicle. *P* value calculated by one-sided Student’s *t*-test. **f**–**h**, Lifespan of mice treated with or without PAM: female mice (**f**), male mice (**g**) and mixed sexes (**h**). Data represent the mean ± s.e.m. log-rank (Mantel–Cox) test was used for survival analyses. Source Data

We next investigated in vivo effect of PAM treatment. To that end, we treated 22-month-old female mice with PAM (1 mg kg^−1^ body weight) or vehicle (phosphate-buffered saline, PBS) by intraperitoneal injection. Two months after treatment, we found that the number of CD133^+^ ELCs was significantly increased in the PAM-treated mice compared with control mice (Fig. 6a). The senescence marker genes *p16* and *Il-6* were both significantly downregulated in CD133^+^ ELCs isolated from the PAM-treated mice (Fig. 6b). We also investigated whether PAM treatment enhanced the function of CD133^+^ ELCs. To this end, we treated 18-month-old female mice with PAM (1 mg kg^−1^) every week for 2 months. Then, CD133^+^ ELCs were isolated from both treated/control mice by FACS, and a total 1 × 10^6^ of them were injected into FAL model via tail vein on day 1 and day 7 after surgery. Indeed, the blood flow recovery rate was significantly enhanced in mice injected with ELCs isolated from the PAM-treated mice compared with those from the control-treated mice (Fig. 6c).

We next explored whether inhibiting FDPS with long-term PAM treatment can rejuvenate aged tissues and promote longevity in mice. We divided a cohort of old female mice, aged around 90 weeks (~21 months), into two groups that were treated with PAM (1 mg kg^−1^) or vehicle (PBS) by intraperitoneal injection. The health status and lifespan of the groups were closely monitored. Six months after PAM treatment, the capillary density (CDH5^+^ ECs) in the heart tissues was significantly increased, from 1,610.24 ± 131.32 mm^−^^2^ to 1,889.3 ± 135.6 mm^−^^2^ (*P* < 0.05) (Fig. 6d). Moreover, mice treated with PAM showed a faster active-to-rest shift than those untreated during the dark-to-light transition (Fig. 6e). For mice that lived longer than median survival time (~115 weeks in vehicle; ~122 weeks in PAM-treated), the lifespan of PAM-treated female mice was significantly extended from 118.5 ± 4.3 weeks to 128.4 ± 3.52 weeks (Fig. 6f, *P* = 0.0371). Likewise, we investigated the effect of PAM treatment in male mice. Interestingly, both median and maximum lifespan were significantly increased in male mice treated with PAM (Fig. 6g, *P* = 0.02). Significant lifespan extension was also noticed if the two sexes were combined (Fig. 6h, *P* = 0.0304). Thus, we concluded that PAM treatment can extend lifespan in mice.

## Discussion

The stem-cell theory of aging dictates that a decline in stem-cell number and/or function leads to defective tissue regeneration and consequential organismal aging. The rapid depletion of adult stem cells resulting from replicative stress caused by somatic cell loss leads to premature aging^40,41^. HSCs and MSCs are two stem-cell populations found in the BM that have great clinical potential. We have previously shown that the number and function of MSCs and HSCs decline in the progeria mouse model, *Zmpste24*^*−/−*^ mice^42^. Here we identified CD133^+^ ELCs as potential EPCs in the BM, and consistent with the decline in HSCs and MSCs, the number and function of CD133^+^ ELCs decreased in progeria mice. During physiological aging, while the number of CD133^+^ ELCs was not much changed, their function in neovascularization was substantially compromised in mice. Remarkably, CD133^+^ ELC rejuvenation improved the microvasculature, attenuated body weight loss and osteoporosis, and extended the lifespan of both progeria and physiologically aged mice. Our findings provide solid evidence to support the use of stem-cell therapy for progeria and normal aging.

The nomenclature and origin of EPCs are controversial^10^. With the benefits of single-cell transcriptome techniques, studies have been able to define various cell populations in BMNCs and BM niche ECs^35,36^. By pseudo-time analyses, we found that CD133-marked BMNCs are closely related to ECs in the BM. Using genetically engineered ROSA^mT/mG^ mouse model, we demonstrated that CD133^+^ ELCs have EC-differentiation and neovascularization ability. We noticed that, in the pseudo-time axis, CD133^+^ ELCs (cluster SC_2) moderately expressed CD14 and VCAM1 but barely expressed CD34, CD105, CD146, CD31 and VE-cadherin, which mark ECs and ECFCs. ECs lost the expression of CD133, and cluster SC_1 expressed moderate levels of CD14, CD34, and ICAM1 (Fig. 2d). Previously, Peichev et al. found that co-expression of CD133 and VEGFR2 identifies circulating endothelial precursors^43^. We found that VEGFR2 was highly expressed in CD133^+^ ELCs. In contrast to MACs and ECFC^14^, we failed to expand CD133^+^ ELCs in vitro. From the compiled evidence, we reasoned that CD133 probably marks an early stage during EPC evolution. CD133^+^ ELCs can be clustered into four groups according to the single-cell transcriptomics, of which CD133_1 was most closely related to the BM ECs, marked with high level of MPO, while CD133_4 was extensively labeled with MMP9. Most importantly, cluster CD133_1 labeled by CD133^+^ and MPO^+^ dictated the main function of CD133^+^ ELCs in neovascularization and lifespan extension. On the other side, we speculated that the cluster CD133_4, that is, CD133^+^MMP9^+^ BMNCs, is more closely related to MACs, which rather elicit paracrine effects during angiogenesis. Consistent with our findings, Kanayasu-Toyoda et al. showed that CD133^+^MMP9^+^ cells in human peripheral blood promote angiogenesis in a paracrine manner^44^. In contrast, the clusters CD133_2 and CD133_3, that is, CD133^+^MPO^−^MMP9^−^ BMNCs, showed the least neovascularization and lifespan-extending capacity. Further in-depth studies are needed to understand the differential and precise roles of CD133^+^ subclusters in neovasculogenesis and aging.

We discovered that BM-derived CD133^+^ ELCs gave vascular-regeneration capacity in the hearts, muscles and blood vessels but not in the livers and kidneys. It is plausible that residential EPCs may also exist in specific tissues/organs, where they mediate the homeostasis of the VE. In the context of aging, ELC replenishment substantially elevates capillary density in the hearts and muscles. For the translational purpose, it is important to validate the findings in human BM in future study.

Although the number of CD133^+^ ELCs increases during aging, their function declines. Via single-cell profiling, we showed the FDPS-centered pathway that regulates sterol metabolism is essential for ELC function. FDPS regulates biosynthesis of isoprenoids, dolichols, ubiquinones and cholesterol and thus mediates protein farnesylation and/or geranylgeranylation^38^. Here we demonstrated that inhibition of FDPS by PAM enhances the regeneration capacity of CD133^+^ ELCs. Gene expression manipulation of *Fdps* via an rAAVDJ vector accelerated ELC aging and dysfunction. Markedly, in vivo administration of PAM rescued ELC aging, improved their neovascularization ability, and promoted healthiness and lifespan. Interestingly, the HGPS causal factor Prelamin A is farnesylated, and the farnesyltransferase inhibitor can ameliorate premature aging by inhibiting Prelamin A farnesylation and abnormal localization^45–47^. A recent study showed that inhibition of FDPS by PAM rescued premature senescence^48^. Upstream, HMG-CoA reductase catalyzes mevalonate production and hence is required in the production of geranyl pyrophosphate^49^. Statins can inhibit HMG-CoA to mobilize EPCs from the BM to peripheral circulation and enhance endothelial repair^50^. Our data highlight an essential role of the FDPS-mediated sterol metabolic pathway in the regulation of aging and longevity. The detailed mechanism that regulates FDPS expression during aging and how FDPS decline compromises the function of CD133^+^ cells merit further investigation.

Collectively, our findings revealed that, in the BM, CD133 marks ELCs, which have neovascularization potential. Healthy and young CD133^+^ ELCs could rejuvenate aged blood vessels, ameliorate aging features and promote longevity in both progeria and physiologically aged mice. At the molecular level, our data highlight a critical role of the FDPS-centered sterol metabolic pathway in regulating ELC aging/dysfunction and longevity. Screening for chemicals that target the FDPS pathway might be applied to benefit health and longevity. Also implicated by our findings is the potential of using stem-cell-based therapeutic strategies for progeria and in anti-aging applications.

## Methods

### Animals

The *Lmna*^G609G/G609G^, *Lmna*^f/f^;TC and Tie2–cre mice were as previously described^37^. ROSA^mT/mG^ mice were provided by Dr Jian Chen (Suchow University, China). C57BL/6 mice, H11-mG/mR mice and Cdh5-cre/ERT mice were purchased from GemPharmatech. All mice were housed and handled in accordance with protocols approved by the Committee on the Use of Live Animals in Teaching and Research of Shenzhen University.

### Hind limb ischemia

Hind limb ischemia was achieved by unilateral FAL as previously described^51^. Briefly, mice were anesthetized with 2% isoflurane inhalation with an isoflurane delivery system (EZVET). The neurovascular pedicle was visualized under a light microscope following a 1-cm incision in the skin of the left hind limb. Ligations were made in the left femoral artery proximal to the superficial epigastric artery branch and anterior to the saphenous artery. Then, the femoral artery and the attached branches between ligations were excised. The skin was closed using a 4–0 suture line, and erythromycin ointment was applied to prevent wound infection after surgery. Recovery of the blood flow was evaluated before and after surgery using a dynamic microcirculation imaging system (Teksqray). Relative blood flow recovery is expressed as the ratio of ischemia to non-ischemia. At least three mice were included in each experimental group.

### RNA isolation and qPCR analysis

Cells were lysed with TRIzol reagent RNAiso Plus (Takara), and total messenger RNA was isolated according to the manufacturer’s instructions. Reverse transcription was performed with 5× Evo M-MLV RT Master Mix (Accurate Biology) to obtain complementary DNA. Gene expression levels were determined by quantitative PCR (qPCR) with the Hieff qPCR SYBR Green Master Mix (Yeasen) on a CFX Connected Real-Time PCR Detection System (Bio-Rad). All primer sequences are listed in Supplementary Table 2.

### Immunofluorescence staining

Frozen sections (10 µm thickness) of mouse tissues were fixed in 4% paraformaldehyde for 15 min, permeabilized with 0.3% Triton X-100 for 15 min, blocked with 5% bovine serum albumin for 30 min, and incubated with an appropriately diluted primary antibody at 4 °C overnight. After removing the primary antibody by washing thrice with PBS, tissue sections were incubated with Alexa Fluor-conjugated secondary antibody at room temperature for 1 h. After three washes with PBS, the sections were mounted with anti-fade mounting medium with 4′,6-diamidino-2-phenylindole (DAPI). Then images were captured under a DragonFly confocal imaging system (Andor) and analyzed with Imaris Viewer software (Bitplane). All antibodies are listed in Supplementary Table 3.

### Echocardiography

Transthoracic echocardiography (IU22, Philips) was performed after inhalation of isoflurane gas by mice in the different groups. Heart rate, cardiac output, left ventricular posterior wall size, left ventricular end-diastolic size, left ventricular end-systolic diameter, left ventricular ejection fraction and left ventricular fraction shorting were obtained by echocardiographic analysis. At least three mice were included in each experimental group.

### Bone density determination

The thigh bones of mice were collected after euthanasia and fixed with 4% paraformaldehyde at 4 °C overnight. The relevant data were collected using micro-computed tomography (micro-CT) scanning (Scanco Medical, µCT100). Micro-CT parameters used for evaluation were trabecular number (1/mm), trabecular separation/spacing (SP) (mm) and BV/TV (%). Each group included at least three mice.

### Endurance running test

Fatigue resistance was monitored using a rotating-rod treadmill (YLS-4C, Jinan Yiyan Scientific Research Company). Mice were placed on a rotating channel, and the rotation rate was gradually increased to 40 rounds min^−1^. When the mice were exhausted and dropped from the rotating channel, the endurance period was recorded. At least three mice were included in each experimental group.

### BM transplantation

BMNCs from ROSA^mT/mG^ mice or H11-mG/mR mice at 3 months of age were collected and injected via the tail vein into 3-month-old C57BL/6 recipient mice irradiated with a lethal dose of X-rays (9 Gy). In brief, the mice were killed, and the femora and tibiae were separated, cut open at the two ends, and placed in a 0.5-ml micro-centrifuge tube that had a hole drilled in the bottom. A 1.5-ml micro-centrifuge tube was used to nest the 0.5 ml tube, and the pair of tubes were centrifuged at 3,000*g* for 15 s. Red blood cells were removed by gradient density centrifugation (Histopaque-1083, Sigma) at 450*g* for 30 min. BMNCs were resuspended in pre-cooled PBS supplemented with 1% fetal bovine serum, and 1.5 × 10^7^ of collected BMNCs were injected into irradiated recipient mice via the tail vein. After 4 weeks’ recovery, the recipient mice were further subjected to unilateral FAL. Three weeks after FAL, the mice were killed for investigation.

### MACS and cell therapy

BMNCs were obtained as described above. After centrifugation at 450*g* at 4 °C for 5 min, the BMNCs were suspended in 80 μl MACS buffer and incubated with 20 μl anti-CD133 antibody (Miltenyi Biotec) for 15 min, followed by incubation with 20 μl of MACS anti-biotin-microBeads (Miltenyi Biotec) in 80 μl MACS buffer. CD133^+^ ELCs were then obtained by magnetic selection. For ELC therapy, 1 × 10^6^ CD133^+^ ELCs collected from ROSA^mT/mG^ mice were injected via the tail vein into aged mice every other week.

### FACS

BMNCs were obtained as previously described. After centrifuging at 450*g* and 4 °C for 5 min, the cells were suspended in 600 μl MACS buffer and incubated with 5 μl APC-CD133 antibody (BioLegend) at 4 °C for 30 min. CD133^+^ EPCs were then sorted on a flow cytometer (BD Biosciences), and data were analyzed with FlowJo software (Becton Dickinson).

### PAM treatment

PAM (Selleckchem) was dissolved in PBS and intraperitoneally injected into female mice at a dose of 3 mg kg^−1^ weekly, beginning at around 90 weeks of age. The health status and lifespan of the mice were closely monitored. Eight weeks after treatment, heart function and running wheel activity were determined.

### Ex vivo Matrigel plug assay

The angiogenic ability of CD133^+^ ELCs was determined with an ex vivo Matrigel plug assay. Briefly, 500 μl of growth factor-reduced Matrigel (Corning) together with 5 × 10^5^ CD133^+^ ELCs or CD133^‒^ ELCs were injected subcutaneously into the middorsal region of 12-week-old male mice. After 2 weeks, the Matrigel plugs were surgically excised, then frozen sections were prepared, and an immunofluorescence assay was performed.

### Single-cell library preparation, sequencing and mapping

CD133^+^ ELCs (approximately 8,000–10,000 cells) were enriched by FACS for single-cell sequencing. RNA was quantified by qubits and PCR with reverse transcription on the LightCycler 96 system (Roche Life Science). The single-cell RNA was barcoded, reverse transcribed using the Chromium Single Cell 3′ Reagent Kit v2 (10x Genomics), fragmented, and amplified to generate cDNA. The Agilent high-sensitivity D5000 and D1000 ScreenTape systems (Agilent) were used to detect the size of pre-amplified cDNA. The sequence libraries were built according to the manufacturer’s instructions, and single-cell libraries were sequenced in a single-index customized paired-end format on the HiSeq 1500 system (Illumina). The data were compared in STAR. According to the reads mapped to the reference genome, we obtained the statistics of specific unique molecular identifier and the expression of each gene in each cell using Cell Ranger 2.1.0 (mean reads per cell 74,323–92,229, and median genes per cell range 1,947–2,342).

### Data quality control and cell-type identification

We mapped each data matrix using the Seurat software package (v3.0.1) and performed data processing and visualization with custom scripts in R. Genes detected in fewer than three cells and cells expressing fewer than 200 genes were removed. Seurat FindVariableFeatures was applied to identify highly variable genes. After deleting for batch effects, the recombined dataset was filtered according to the following criteria to exclude inferior cells: unique molecular identifier count <5% or >95% or the proportion of mitochondrial genes >20%. Principal component analysis was used to perform clustering through FindNeighbors and FindClusters functions. We used *t*-distributed random neighbor embedding to reduce the dimensionality and visualize the clustering results. The cell type results identified by the singleR package were used as a reference, and the marker genes of the cell cluster were used to identify the cell type.

### Single-cell data analysis

Unsupervised pseudo-time analysis of single cells was performed on Monocle software, and the results were used as a reference for the differentiation process of BM cells. Seurat FindMarkers function and MAST package (v1.8.2) were used to calculate differentially expressed genes for each cell type with a false discovery rate-adjusted *P* value of <0.05. The protein–protein interaction network functional enrichment analysis was based on the STRING database (https://string-db.org/) and visualized by Cytoscape (V3.6.1). The gene set enrichment analysis was based on the enrichKEGG and enrichGO functions of the clusterProfiler package and the GSEA package.

### Statistics and reproducibility

Statistical data were obtained with Excel (Microsoft 365 Family), and low-throughput statistical analysis was performed using GraphPad Prism (v9.2.0). Two-tailed Student’s *t*-test was used to determine the statistical significance between two groups of data. One-way analysis of variance (ANOVA) analysis was used to test the statistical significance between multiple groups of data. Two-way ANOVA analysis was used to test the statistical significance between multiple sets of data of time series. log-rank (Mantel–Cox) test was used for survival analyses. All data are presented as the mean ± standard deviation or mean ± standard error of the mean (s.e.m.) as indicated, and a *P* value of <0.05 was considered statistically significant. Data distribution was assumed to be normal, but this was not formally tested. For in vivo animal studies, a minimum of four samples were included, and animals were randomly assigned to different experimental groups. The in vitro assay involved a minimum of three samples, each repeated three times, and no animals or data points were excluded from the analyses for any reason. In this study, the experimenter was blinded to the grouping of all mouse experiments. Data distribution was assumed to be normal, but this was not formally tested.

### Reporting summary

Further information on research design is available in the Nature Portfolio Reporting Summary linked to this article.

### Supplementary information

Supplementary Information Supplementary Figs. 1–6.

Reporting Summary

Supplementary Tables 1–3 Gene list, primer list and antibody list.

Supplementary Video 1 3D reconstruction of immunofluorescence images of heart section.

Supplementary Video 2 3D reconstruction of immunofluorescence images of kidney section.

Supplementary Video 3 3D reconstruction of immunofluorescence images of muscle section.

Supplementary Video 4 3D reconstruction of immunofluorescence images of liver section.

Supplementary Video 5 3D reconstruction of immunofluorescence images of heart section.

Supplementary Video 6 3D reconstruction of immunofluorescence images of muscle section.

### Source data

Source Data Fig. 1 Statistical source data.

Source Data Fig. 2 Statistical source data.

Source Data Fig. 3 Statistical source data.

Source Data Fig. 4 Statistical source data.

Source Data Fig. 5 Statistical source data.

Source Data Fig. 6 Statistical source data.

Source Data Extended Data Fig. 1 Statistical source data.

Source Data Extended Data Fig. 6 Statistical source data.

## Data Availability

Single-cell RNA sequencing raw fastq files and processed data were deposited and are freely available in the NCBI Gene Expression Omnibus (GSE233944). All other data are available from the corresponding author upon reasonable request. Publicly available data obtained and used in the study were: whole mouse BMNCs (GSE109774) and BM niche CDH5^+^ cells (GSE108892) from the Gene Expression Omnibus (https://www.ncbi.nlm.nih.gov/geo/).
