# Author Correction: CD133^+^ endothelial-like stem cells restore neovascularization and promote longevity in progeroid and naturally aged mice

**DOI:** 10.1038/s43587-023-00543-6

**Published:** 2023-12-11

**Authors:** Shimin Sun, Yuan Meng, Mingying Li, Xiaolong Tang, Wenjing Hu, Weiwei Wu, Guo Li, Qiuxiang Pang, Wengong Wang, Baohua Liu

**Affiliations:** 1 Shenzhen Key Laboratory for Systemic Aging and Intervention (SKL-SAI), Guangdong Key Laboratory of Genome Stability and Human Disease Prevention; International Cancer Center, School of Basic Medical Sciences, Shenzhen University, Shenzhen, China; 2 Friedrich Schiller University, Jena, Germany; 3 Wannan Medical College, Wuhu, China; 4 School of Biomedical Sciences, Hunan University, Changsha, China; 5 Anti-aging & Regenerative Medicine Research Institution, School of Life Sciences, Shandong University of Technology, Zibo, China; 6 Department of Dermatology, Xiangya Hospital, Central South University, Changsha, China; 7 Department of Biochemistry and Molecular Biology, School of Basic Medical Sciences, Peking University Health Science Center, Beijing, China

**Keywords:** Ageing, Senescence, Ageing

Correction to: *Nature Aging* 10.1038/s43587-023-00512-z, published online 9 November 2023.

In the version of the article initially published, the Data Availability section was incorrect and has been corrected to read “All sequencing data generated for this study is available at Array Express with following accession numbers: Spatial Transcriptomics from young and old liver: E-MTAB-12809. scATAC-seq of young and old livers: E-MTAB-12706 and E-MTAB-12560. SMART-seq3xpress data on young and old hepatocytes: E-MTAB-12579. H3K27ac for young and old mice was downloaded from BioProject PRJNA281127. Tabula Muris senis single cell data is available at Gene Expression Omnibus GSE149590. Tabula Muris senis bulk RNA-seq data is available at Gene Expression Omnibus GSE132040. All other data will be provided by the corresponding author upon reasonable request.”

