# Supplementary material for: CD133+ endothelial-like stem cells restore neovascularization and promote longevity in progeroid and naturally aged mice
Source: Nat Aging. 2023 Nov 9;3(11):1401–14. doi: 10.1038/s43587-023-00512-z (PMC10645602; doi:10.1038/s43587-023-00512-z)
Supplement: Supplementary file 1 — Supplementary Figs. 1–6. [file 43587_2023_512_MOESM1_ESM.pdf]

nature aging

Article

https://doi.org/10.1038/s43587-023-00512-z

CD133^{+} endothelial-like stem cells restore neovascularization and promote longevity in progeroid and naturally aged mice

In the format provided by the authors and unedited

Table of Contents

Fig. S1 Donor-derived ECs are barely detectable in livers, lungs, and kidneys. 3

Fig. S2 FACS and microscopy analysis of BMTed cell differentiation labeled by AAV-mediated Cre expression 5

Fig. S3 Donor and recipient cell fusion was excluded. 7

Fig. S4 Gene expression profile in CD133$^+$ ELCs. 8

Fig. S6 Pathway analysis of differentially expressed genes between young and old CD133$^+$ ELCs 10

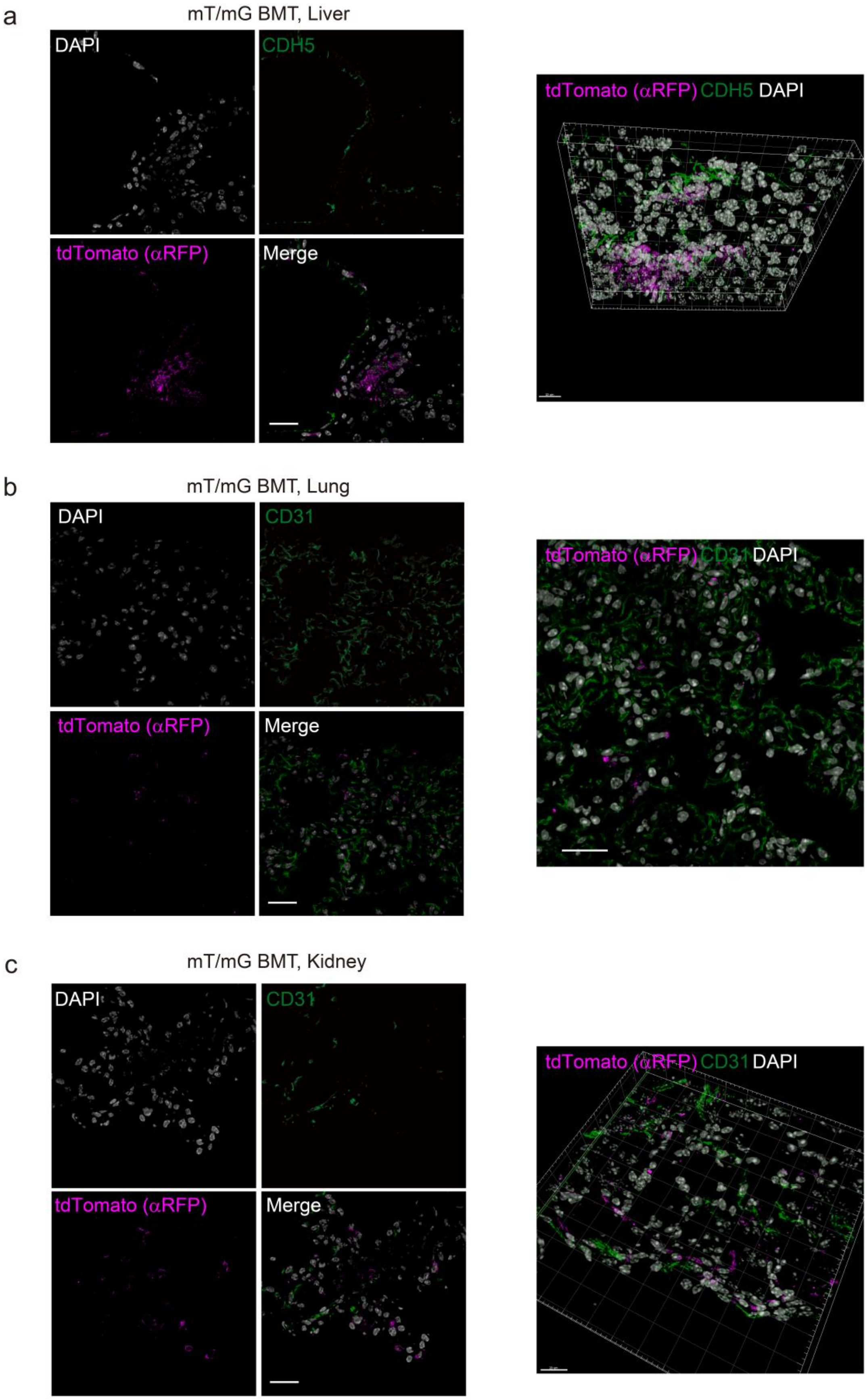
a
mT/mG BMT, Liver
DAPI
CDH5
tdTomato (αRFP)
Merge
tdTomato (αRFP) CDH5 DAPI
b
mT/mG BMT, Lung
DAPI
CD31
tdTomato (αRFP)
Merge
tdTomato (αRFP) CD31 DAPI
c
mT/mG BMT, Kidney
DAPI
CD31
tdTomato (αRFP)
Merge
tdTomato (αRFP) CD31 DAPI

Fig. S1 Donor-derived ECs are barely detectable in livers, lungs, and kidneys.

(**a**) Representative fluorescence images showing donor-derived $CDH5^+$ and RFP^+ ECs in liver tissues.

(**b-c**) Representative images showing donor-derived $CD31^+$ and RFP^+ ECs in lungs (**b**) and kidneys (**c**).

Scale bar, 20 μm. 3 mice were analyzed.

Pseudocolor scheme used in immunofluorescent images: white represents the nucleus stain (DAPI), and magenta represents red signal (tdTomato(RFP) labelled with Alexa Fluor 594 conjugated antibodies).

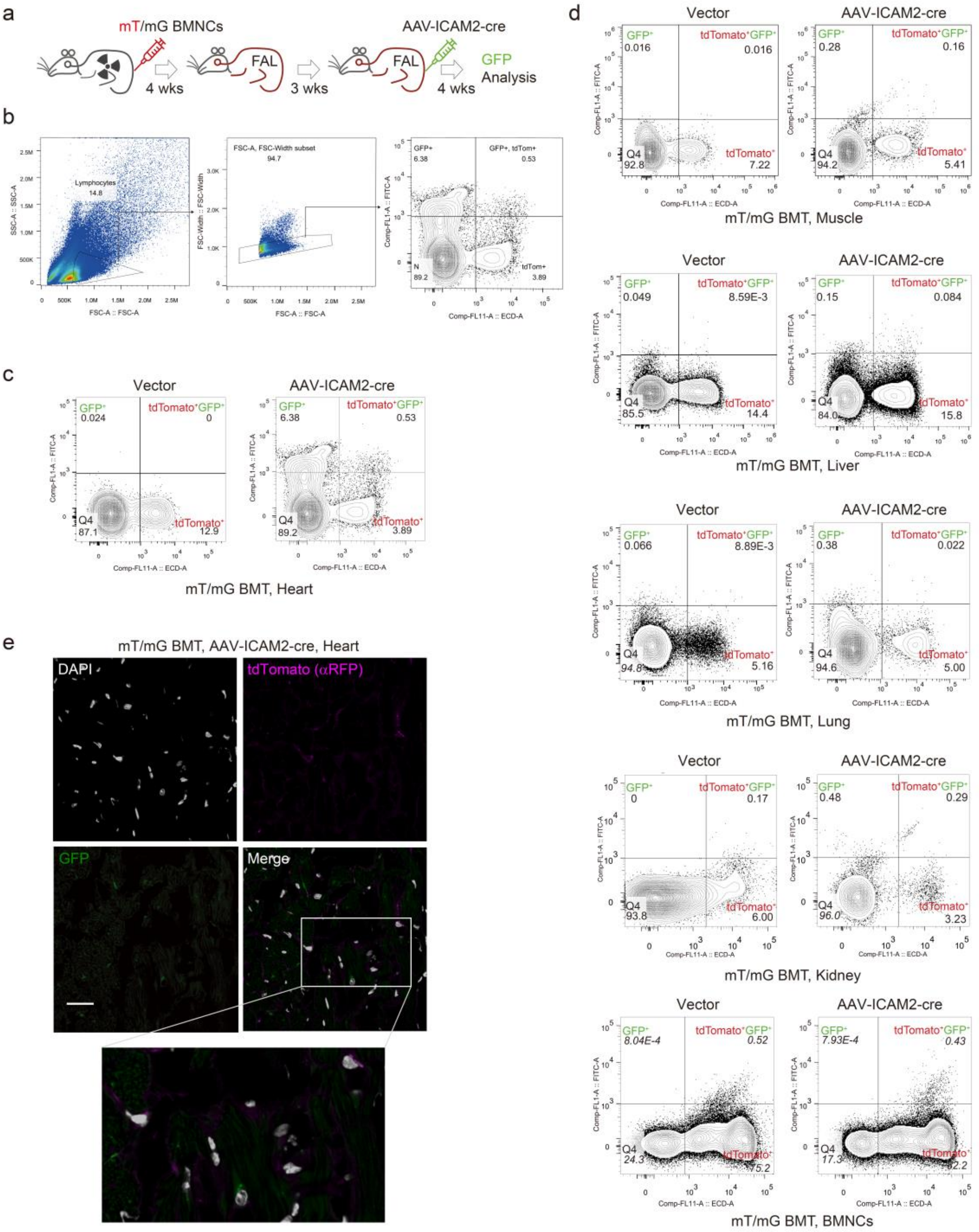
a
mT/mG BMNCs
4 wks
FAL
3 wks
AAV-ICAM2-cre
4 wks
GFP
Analysis
b
Lymphocytes
14.8
FSC-A, FSC-Width subset
94.7
GFP+
6.38
GFP+, tdTom+
0.53
tdTom+
3.89
89.2
c
Vector
AAV-ICAM2-cre
GFP+
tdTomato+GFP+
tdTomato+
Q4
mT/mG BMT, Heart
d
Vector
AAV-ICAM2-cre
mT/mG BMT, Muscle
mT/mG BMT, Liver
mT/mG BMT, Lung
mT/mG BMT, Kidney
mT/mG BMT, BMNCs
e
mT/mG BMT, AAV-ICAM2-cre, Heart
DAPI
tdTomato (αRFP)
GFP
Merge

Fig. S2 FACS and microscopy analysis of BMTed cell differentiation labeled by AAV-mediated Cre expression

(**a**) Schematic of lineage-tracing strategy: recipient mice were irradiated with X-rays and transplanted with BMNCs from $ROSA^{mT/mG}$ mice; after 4 weeks, the recipient mice were subjected to unilateral FAL; after another 3 weeks, the AAV1-ICAM2-cre particles were injected via tail vein. After 4 weeks, mice were euthanized and tissues were collected for flow cytometry analysis.

(**b**) FACS gating strategy

(**c**) FACS analysis of fluorescence cells in heart tissues from $ROSA^{mT/mG}$ BMTed mice, treated with AAV-ICAM2-cre or AAV particles. FITC, for GFP signals; ECD, for tdTomato signals.

(**d**) FACS analysis of fluorescence cells in muscle, liver, lung and kidney tissues from $ROSA^{mT/mG}$ BMTed mice, treated with AAV-ICAM2-cre or AAV particles. FITC, for GFP signals; ECD, for tdTomato signals.

(**e**) Representative fluorescence images showing donor derived GFP^+, $tdTomato^+$ and $GFP^+tdTomato^+$ cells in heart sections from $ROSA^{mT/mG}$ BMTed mice treated with AAV-ICAM2-Cre viral particles.

5 mice were analyzed.

Pseudocolor scheme used in immunofluorescent images: white represents the nucleus stain (DAPI), and magenta represents red signal (tdTomato(RFP) labelled with Alexa Fluor 594 conjugated antibodies).

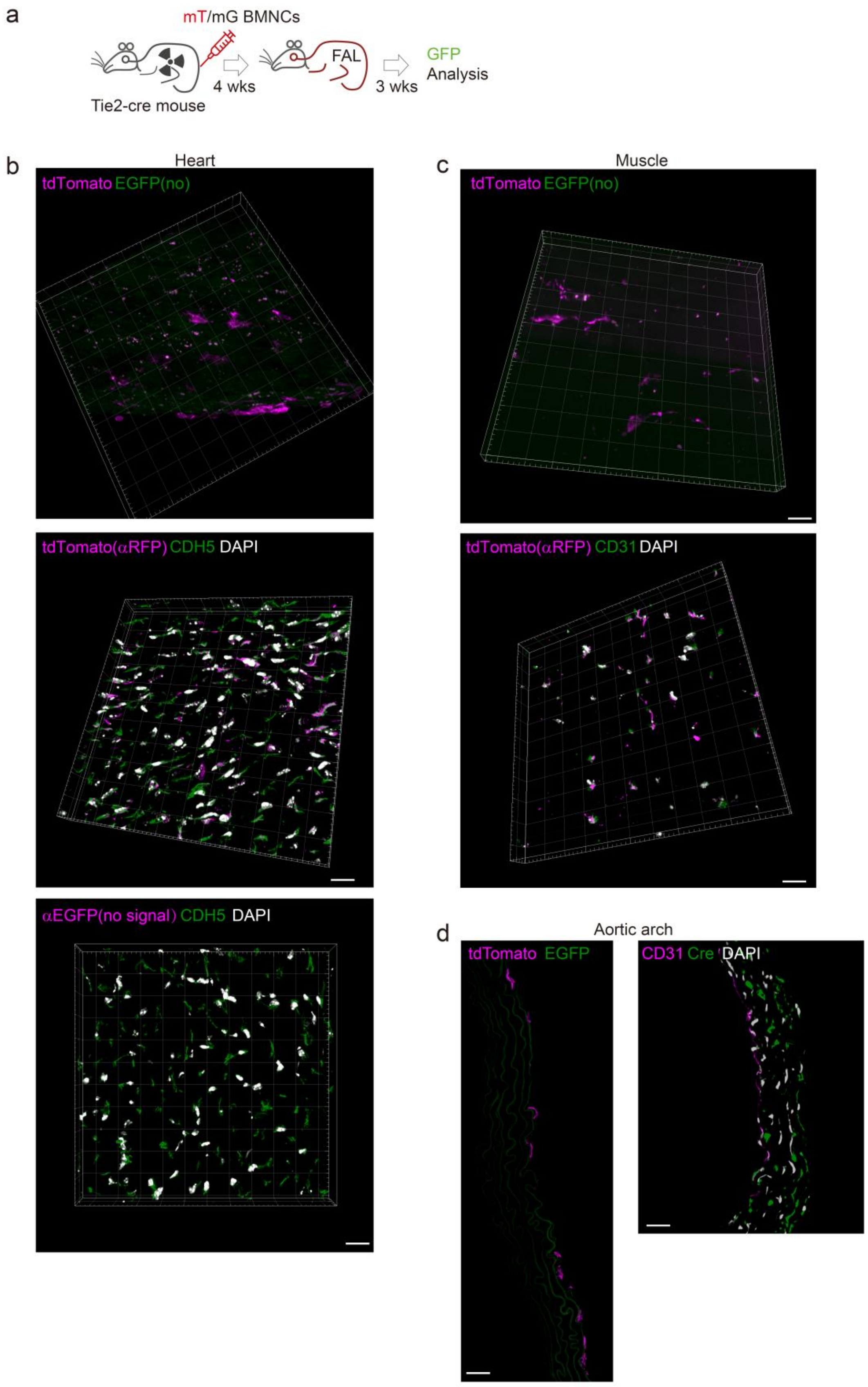
a
mT/mG BMNCs
4 wks
FAL
3 wks
GFP
Analysis
Tie2-cre mouse
b
Heart
tdTomato EGFP(no)
tdTomato(αRFP) CDH5 DAPI
αEGFP(no signal) CDH5 DAPI
c
Muscle
tdTomato EGFP(no)
tdTomato(αRFP) CD31 DAPI
d
Aortic arch
tdTomato EGFP
CD31 Cre DAPI

Fig. S3 Donor and recipient cell fusion was excluded

(**a**) Schematic of lineage-tracing strategy: The Tie2-cre recipient mice were irradiated with X-rays and transplanted with BMNCs from ROSA$^{mT/mG}$ mice; 4 weeks after BMT, the recipient mice were subjected to unilateral FAL. Three weeks after FAL, the mice were sacrificed for investigation.

(**b**) Representative fluorescence images showing co-staining of anti-RFP (tdTomato) and EGFP (upper, no EGFP detected), anti-RFP and CDH5 (middle), and anti-EGFP and CHD5 (lower, no EGFP detected) in heart tissue isolated from irradiated recipient Tie2-cre mice BMTed with rosa26-mT/mG BMNCs. Scale bar, 20 µm. 3 mice were examined.

(**c**) Representative fluorescence images showing co-staining of anti-RFP (tdTomato) and EGFP (upper, no EGFP detected) and anti-RFP and CD31 (lower) in muscle isolated from irradiated recipient Tie2-cre mice BMTed with ROSA$^{mT/mG}$ BMNCs. Scale bar, 20 µm. 3 mice were examined.

(**d**) Representative images showing tdTomato and EGFP fluorescence (left, no EGFP detected) and co-staining of anti-cre and CD31 (right) in aortic arch of Tie2-cre mice BMTed with ROSA$^{mT/mG}$ BMNCs. Scale bar, 100 µm. 3 mice were examined.

Pseudocolor scheme used in immunofluorescent images: white represents the nucleus stain (DAPI), and magenta represents red signal (tdTomato live fluorescence signal or labeled with Alexa Fluor 594 conjugated antibodies, EGFP/CD31 labelled with Alexa Fluor 594 conjugated antibodies).

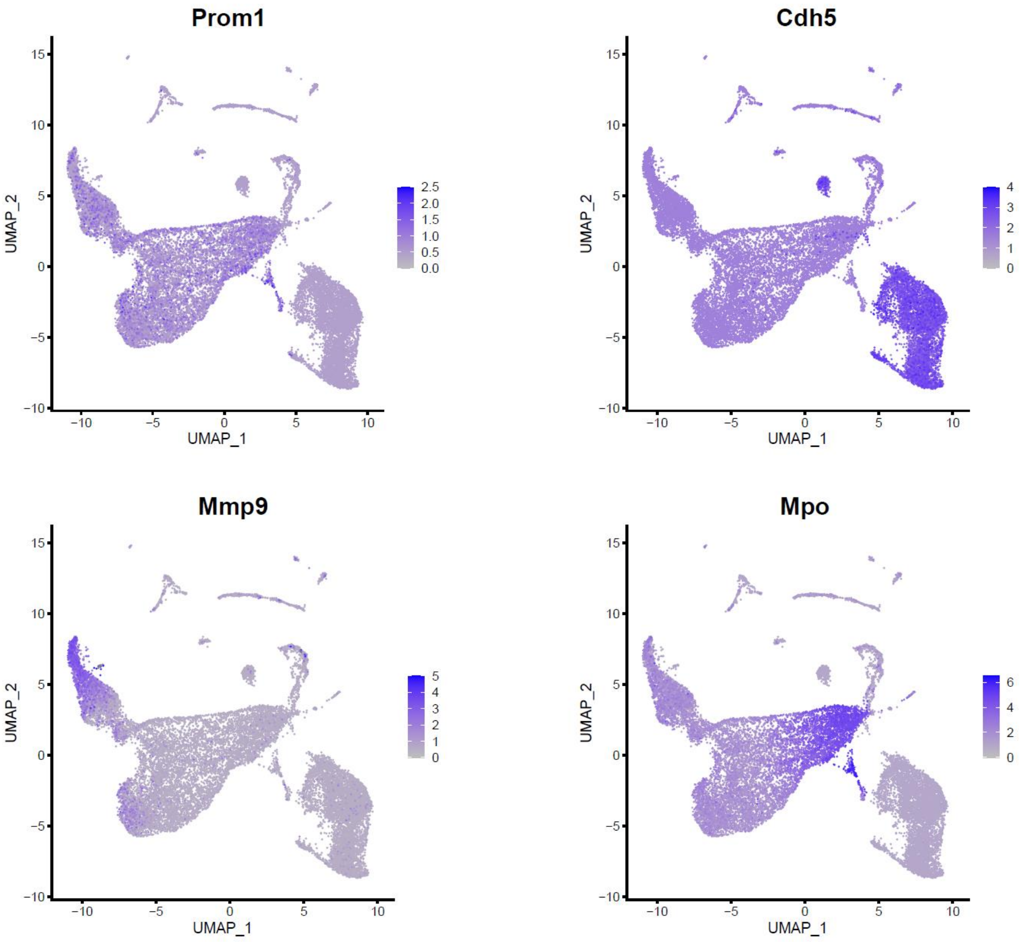

Fig. S4 Gene expression profile in CD133^{+} ELCs**.**

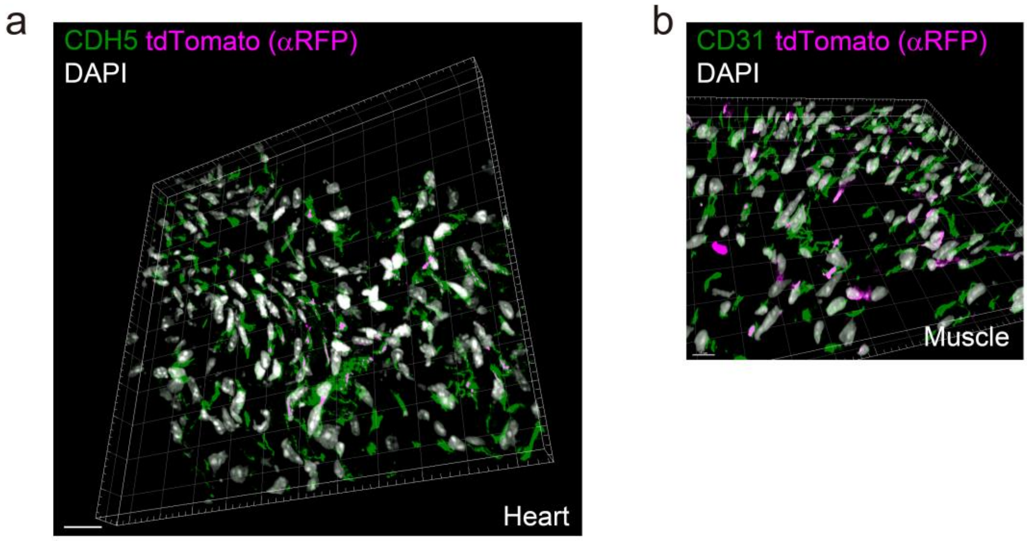

Fig. S5 3D visualization of co-immunofluorescence-stained ECs

(**a**-**b**) 3D visualization of co-staining for CDH5 and RFP (tdTomato) in heart (a) and CD31 and RFP in muscle (b). Scale bar, 20 µm. 3 mice were examined.

Pseudocolor scheme used in immunofluorescent images: white represents the nucleus stain (DAPI), and magenta represents red signal (tdTomato(RFP) labelled with Alexa Fluor 594 conjugated antibodies).

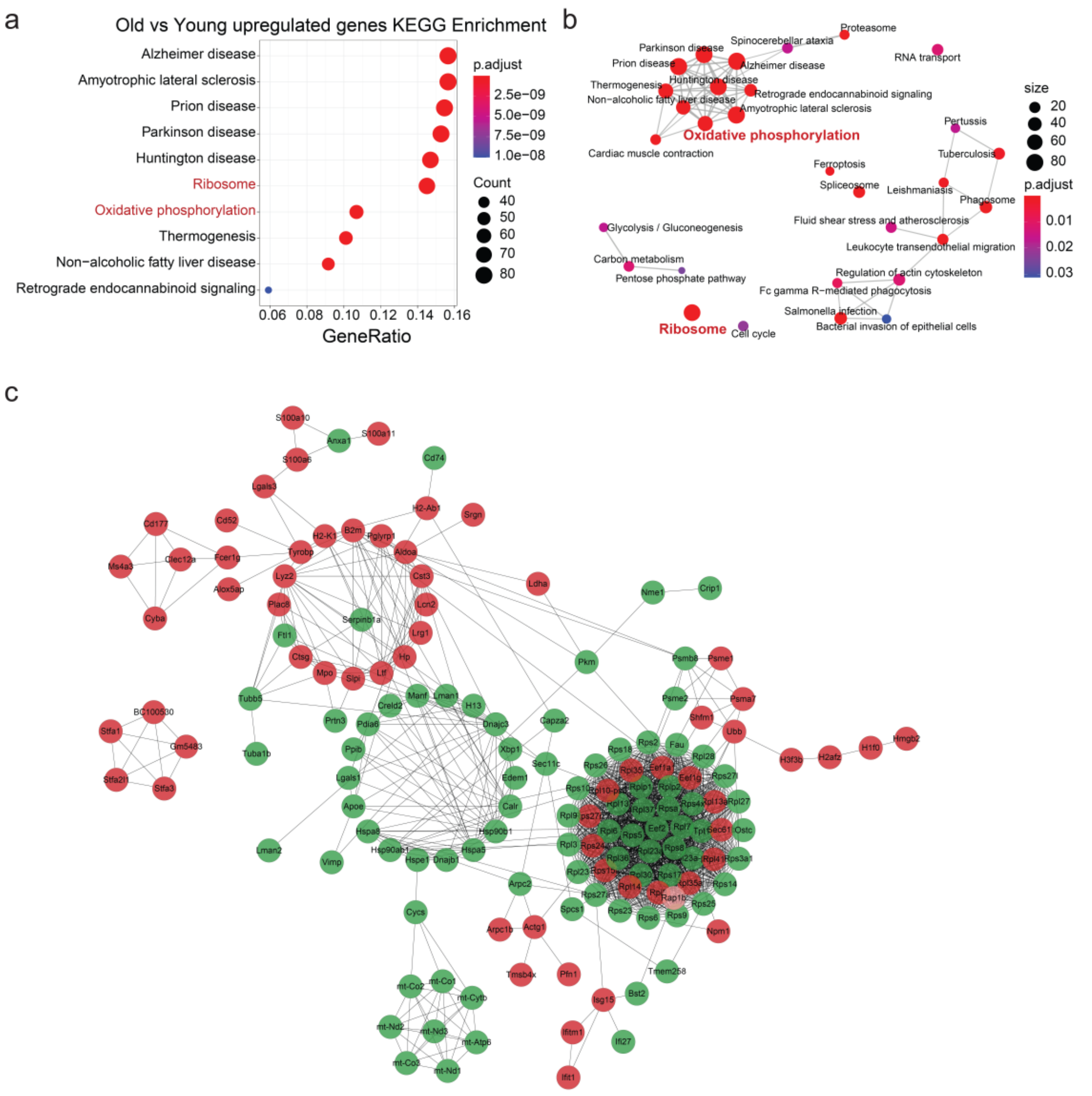

Fig. S6 Pathway analysis of differentially expressed genes between young and old CD133+ ELCs

(**a**) KEGG pathway enrichment of upregulated genes in old CD133+ ELCs.

(**b**) Network diagram showing pathways with overlapping genes based on GO and KEGG enrichment analysis of upregulated genes in old CD133+ ELCs.

(**c**) Protein-protein interaction network of top-200 differentially expressed genes in the ribosome and oxidative phosphorylation pathways enriched in (**a**) and (**b**).

Hypergeometric distribution methods are used to determine whether there is enrichment in the list of differentially expressed genes of known biological pathways. Use the Benjamini-Hochberg correction method to compute and adjust the positive p-values.
