# Supplementary material for: CD133+ endothelial-like stem cells restore neovascularization and promote longevity in progeroid and naturally aged mice
Source: Nat Aging. 2023 Nov 9;3(11):1401–14. doi: 10.1038/s43587-023-00512-z (PMC10645602; doi:10.1038/s43587-023-00512-z)
Supplement: Supplementary file 2 — Reporting Summary [file 43587_2023_512_MOESM2_ESM.pdf]

Corresponding author(s): Baohua Liu

Last updated by author(s): Aug 25, 2023

Reporting Summary

Nature Portfolio wishes to improve the reproducibility of the work that we publish. This form provides structure for consistency and transparency in reporting. For further information on Nature Portfolio policies, see our Editorial Policies and the Editorial Policy Checklist.

Statistics

For all statistical analyses, confirm that the following items are present in the figure legend, table legend, main text, or Methods section.

n/a	Confirmed	
☐	☒	The exact sample size (*n*) for each experimental group/condition, given as a discrete number and unit of measurement
☐	☒	A statement on whether measurements were taken from distinct samples or whether the same sample was measured repeatedly
☐	☒	The statistical test(s) used AND whether they are one- or two-sided<br>*Only common tests should be described solely by name; describe more complex techniques in the Methods section.*
☒	☐	A description of all covariates tested
☐	☒	A description of any assumptions or corrections, such as tests of normality and adjustment for multiple comparisons
☐	☒	A full description of the statistical parameters including central tendency (e.g. means) or other basic estimates (e.g. regression coefficient) AND variation (e.g. standard deviation) or associated estimates of uncertainty (e.g. confidence intervals)
☒	☐	For null hypothesis testing, the test statistic (e.g. *F*, *t*, *r*) with confidence intervals, effect sizes, degrees of freedom and *P* value noted<br>*Give P values as exact values whenever suitable.*
☒	☐	For Bayesian analysis, information on the choice of priors and Markov chain Monte Carlo settings
☒	☐	For hierarchical and complex designs, identification of the appropriate level for tests and full reporting of outcomes
☒	☐	Estimates of effect sizes (e.g. Cohen's *d*, Pearson's *r*), indicating how they were calculated

Our web collection on statistics for biologists contains articles on many of the points above.

Software and code

Policy information about availability of computer code

Data collection	Real-time PCR data were collected by BIO-RAD CFX Connect.<br>Flow cytometry data were collected by FACS Aria II (BD bioscience, USA).<br>Immunofluorescent images were collected by Zeiss LSM880 confocal microscope (Zeiss Germany)<br>The echocardiographic evaluation data were collected by Philips IU22 Ultrasound Machine (Royal Dutch Philips).<br>The blood flow data were collected by dynamic microcirculation imaging system (Teksqray, Shenzhen, China).<br>The bone density relevant data were collected by micro-CT scanning (Scanco Medical, μCT100).<br>The fatigue resistance data were collected by a rotating-rod treadmill (YLS-4C, Jinan Yiyan<br>Scientific Research Company, China).<br>The single-cell libraries (10×Genomics, USA) were sequenced in a single-index customized paired-end format on the HiSeq 1500 system (Illumina, USA).
Data analysis	Excel (Microsoft 365 Family) and GraphPad Prism 9.2.0 software were used for statistical analyses.<br>FlowJo_v10 software was used for flow cytometry data analyses.<br>Imaris Viewer x649.7.0 software was used for immunofluorescent images analyses.<br>R (version 3.5 - 4.1.2) were used for sequencing data analysis.

For manuscripts utilizing custom algorithms or software that are central to the research but not yet described in published literature, software must be made available to editors and reviewers. We strongly encourage code deposition in a community repository (e.g. GitHub). See the Nature Portfolio guidelines for submitting code & software for further information.

Data

Policy information about availability of data

All manuscripts must include a data availability statement. This statement should provide the following information, where applicable:

- Accession codes, unique identifiers, or web links for publicly available datasets
- A description of any restrictions on data availability
- For clinical datasets or third party data, please ensure that the statement adheres to our policy

All secuencing datasets have been deposited to GEO which wil be made available upon publication using the following acession code: GSE233944

Human research participants

Policy information about studies involving human research participants and Sex and Gender in Research.

Reporting on sex and gender	N/A
Population characteristics	N/A
Recruitment	N/A
Ethics oversight	N/A

Note that full information on the approval of the study protocol must also be provided in the manuscript.

Field-specific reporting

Please select the one below that is the best fit for your research. If you are not sure, read the appropriate sections before making your selection.

☒ Life sciences ☐ Behavioural & social sciences ☐ Ecological, evolutionary & environmental sciences

For a reference copy of the document with all sections, see nature.com/documents/nr-reporting-summary-flat.pdf

Life sciences study design

All studies must disclose on these points even when the disclosure is negative.

Sample size	The sample size was not statistically predetermined. The sample for q-PCR, IF analyses were harvested from at least three biological replicates and the sample size were chosen in agreement with common practice in the field (Wei Wang et al. 2021; YI Bao et al,2020; Stephanie P.et al., 2021).For in vivo animal studies, a minimum of 4 samples were included, and animals were randomly assigned to different experimental groups. The in vitro assay involved a minimum of 3 samples, each repeated 3 times. For the single cell-sequencing, we combined the BMNCs from 3 mice together for sequencing. The mixed individual sample setting for scRNA-seq is widely used (Maria Hurskainen et al. 2021; Inamul Kabir et al. 2023). For the echocardiographic evaluation, bone density determination, endurance running test and lifespan analysis, the sample size was determined on the sample availability (more is better) and the samples sizes were similar to those generally employed and accepted in the field (Burd, C.E.et al.,2013; Zhonghao Zhang et al., 2017). All sample sizes are shown in the figure legends or under the methods section of our manuscript, respectively.
Data exclusions	there is no any animals were excluded from the analyses for any reason.
Replication	All experiments were performed with at least three biological replicates and confirmed from more than two independent experiments.
Randomization	All the treated groups and control groups were randomly assigned, and the images were collected randomly from at least 5 fields for each section.
Blinding	In this study, the experimenter was blinded to the grouping of all mouse experiments.

Reporting for specific materials, systems and methods

We require information from authors about some types of materials, experimental systems and methods used in many studies. Here, indicate whether each material, system or method listed is relevant to your study. If you are not sure if a list item applies to your research, read the appropriate section before selecting a response.

Materials & experimental systems

n/a	Involved in the study
☐	☒ Antibodies
☒	☐ Eukaryotic cell lines
☒	☐ Palaeontology and archaeology
☐	☒ Animals and other organisms
☒	☐ Clinical data
☒	☐ Dual use research of concern

Methods

n/a	Involved in the study
☒	☐ ChIP-seq
☐	☒ Flow cytometry
☒	☐ MRI-based neuroimaging

Antibodies

Antibodies used

1. CD31, Abcam (ab7388), IF (1:150);
2. CDH5(VE-Cadherin), R&D systems (AF1002), IF (1:150);
3. RFP, Abcam (ab62341), IF (1:100);
4. F4/80, Abcam (ab6640), IF (1:100);
5. CD31-FITC, Biolegend (102506), Flow Cyt (1:1000);
6. CD31-PE, BD Bioscience (561410), Flow Cyt (1:1000);
7. CD133-APC, Biolegend (141208), Flow Cyt (1:100);
8. Myeloperoxidase-FITC, Abcam (ab90812), Flow Cyt (1:100);
9. MMP9-AF488, Abcam (ab194314), Flow Cyt (1:100);
10. prominin-1-biotin(CD133), Miltenyi Biotec (130-101-851), cell sorting(1:10).

Validation

1. All these antibodies below are commercially available and all validated by the producers.
CD31, Abcam (ab7388), IF (1:150);
2. https://www.abcam.cn/cd31-antibody-mec-746-ab7388.html;
CDH5(VE-Cadherin), R&D systems (AF1002), IF (1:150);
3. https://www.rndsystems.com/cn/products/mouse-ve-cadherin-antibody_af1002;
RFP, Abcam (ab62341), IF (1:100);
4. https://www.abcam.cn/rfp-antibody-ab62341.html;
F4/80, Abcam (ab6640), IF (1:100);
5. https://www.abcam.cn/f480-antibody-cia3-1-macrophage-marker-ab6640.html;
CD31-FITC, Biolegend (102506), Flow Cyt (1:1000);
6. https://www.biolegend.com/en-us/products/fitc-anti-mouse-cd31-antibody-377;
CD31-PE, BD Bioscience (561410), Flow Cyt (1:1000);
7. https://www.bdbiosciences.com/en-us/products/reagents/flow-cytometry-reagents/research-reagents/single-color-antibodies-ruo/pe-cy-7-rat-anti-mouse-cd31.561410;
CD133-APC, Biolegend (141208), Flow Cyt (1:100);
https://www.biolegend.com/en-us/products/apc-anti-mouse-cd133-antibody-7243?GroupID=BLG9240;
8. Myeloperoxidase-FITC, Abcam (ab90812), Flow Cyt (1:100);
https://www.abcam.com/fitc-myeloperoxidase-antibody-2d4-ab90812.html;
9. MMP9-AF488, Abcam (ab194314), Flow Cyt (1:100);
https://www.abcam.cn/alexa-fluor-488-mmp9-antibody-ep1255y-ab194314.html;
10. https://www.biocompare.com/9776-Antibodies/7059102-Anti-Prominin-1-antibodies;
prominin-1-biotin(CD133), Miltenyi Biotec (130-101-851), cell sorting(1:10).

Antibodies No. 1 to 4 are used for immunofluorescence staining. Each antibody, upon its first use, is compared with an IgG control to confirm its efficacy. Antibodies No. 5 to 9 are utilized for flow cytometric analysis and sorting. Each antibody, when first used, is compared with an Isotype control to verify its efficacy. Antibody No. 10 is employed for cell sorting and, upon its initial use, is compared with an Isotype control to validate its effectiveness.

Animals and other research organisms

Policy information about studies involving animals; ARRIVE guidelines recommended for reporting animal research, and Sex and Gender in Research

Laboratory animals

The LmnaG609G/G609G mice, C57BL6/J background, 3-6 months, males and females
Lmnaf/f;TC mice, C57BL6/J background, 8 months, males
Tie2-cre mice, C57BL6/J background, 3-6 months, males
ROSA-mT/mG mice, provided by Dr Jian Chen (Suchow University, China), C57BL6/J background, 3 months, males and females
C57BL/6 mice, H11-mG/mR mice and Cdh5-cre/ERT mice, purchased from GemPharmatech Co., Ltd. (Jiangsu, China)
C57BL6/J background, 3 months, males and females

Wild animals

No wild animals were used in this study.

Reporting on sex

With the exception of the male mouse model for premature aging, there was no gender preference in this study. Gender unity was attempted to the greatest extent possible within the same experiment in order to ensure the uniqueness of variables.

Field-collected samples

This study did not involve any field-collected samples.

Ethics oversight	Animal experiments were conducted in accordance with ethical and scientific protocols approved by the Committee on the Use of Live Animals in Teaching and Research of Shenzhen University, China.

Note that full information on the approval of the study protocol must also be provided in the manuscript.

Flow Cytometry

Plots

Confirm that:

☒ The axis labels state the marker and fluorochrome used (e.g. CD4-FITC).

☒ The axis scales are clearly visible. Include numbers along axes only for bottom left plot of group (a 'group' is an analysis of identical markers).

☒ All plots are contour plots with outliers or pseudocolor plots.

☒ A numerical value for number of cells or percentage (with statistics) is provided.

Methodology

Sample preparation	For BMNCs:<br>the mice were sacrificed by euthanasia, and the femora and tibiae were separated, cut open at the two ends, and placed in a 0.5-ml micro-centrifuge tube that had a hole drilled in the bottom. A 1.5-ml micro-centrifuge tube was used to nest the 0.5 ml tube, and the pair of tubes were centrifuged at 3000 × g for 15 sec. Red blood cells were removed by gradient density centrifugation at 450 × g for 30 min. BMNCs were resuspended in pre-cooled PBS supplemented with 1% FBS. After centrifuging at 450 × g and 4°C for 5 min, the cells were suspended in 600 µl MACS buffer for flow cytometry analysis.<br><br>For tissues:<br>After mice were euthanized, the tissues were perfused with pre-cold perfusion buffer, then cut into small pieces and put in digestion solution, 37 °C for 40 minutes. After digestion, the cell lysate passed through a 100 um cell strainer and cells we collected were resuspended in MACS buffer for flow cytometry analysis.
Instrument	The flow cytometry was performed by FACS Aria II (BD bioscience, USA).
Software	FlowJo_V10 software was used for flow cytometry data analyses.
Cell population abundance	~10% of bone marrow cells were identified as Cd133 positive bone marrow cells.<br>~1% of bone marrow cells were identified as Cd133 and Mpo double positive bone marrow cells.<br>~3% of bone marrow cells were identified as Cd133 and Mpo and MMP9 triple-positive bone marrow cells.
Gating strategy	Forward scatter area (FSC-A) and side scatter area (SSC-A) was used to sort for cells.<br>For sorting Cd133 positive bone marrow cells, the APC channel was used to gate based on the APC isotype labeled cell sample.<br>For sorting Cd133 and Mpo double-positive bone marrow cells, we employed an additional FITC channel to sort for Mpo positive bone marrow cells before APC channel selection.<br>For sorting Cd133 and Mpo and MMP9 triple-positive bone marrow cells, we employed an additional FITC channel to sort for Mpo and MMP9 positive bone marrow cells before APC channel selection.

☒ Tick this box to confirm that a figure exemplifying the gating strategy is provided in the Supplementary Information.
